# Circ_0030235 knockdown protects H9c2 cells against OGD/R-induced injury via regulation of miR-526b

**DOI:** 10.7717/peerj.11482

**Published:** 2021-11-16

**Authors:** Yuquan Zhang, Shuzhu Liu, Limin Ding, Dawei Wang, Qiangqiang Li, Dongdong Li

**Affiliations:** 1 Department of Gerontology, The First Hospital of Qiqihar, Qiqihar, Heilongjiang, China; 2 Department of Gerontology, Affiliated Qiqihar Hospital, Southern Medical University, Qiqihar, Heilongjiang, China; 3 Department of Library, The First Hospital of Qiqihar, Qiqihar, Heilongjiang, China; 4 Department of Library, Affiliated Qiqihar Hospital, Southern Medical University, Qiqihar, Heilongjiang, China

**Keywords:** Myocardial infarction, Circ_0030235, miR-526b, PI3K/AKT and MEK/ERK pathways

## Abstract

**Backgrounds:**

Acute myocardial infarction (MI) is the common clinical manifestation of coronary heart disease. Circular RNAs (circRNAs) act key roles in cardiomyocytes growth and angiogenesis. However, their functions in MI are not entirely clear. This research intended to investigate the role and underlying mechanisms of circ_0030235 in H9c2 cells.

**Methods:**

H9c2 cells were conducted to oxygen glucose deprivation/reperfusion (OGD/R) inducement to establish the MI model. Circ_0030235 and miR-526b expression was tested and altered by qRT-PCR and transfection. Cell viability, apoptosis and reactive oxygen species (ROS) injury were tested by CCK-8 assay, TUNEL assay kit, and ROS Detection Assay Kit, respectively. Assessment of cell injury-related factors was performed by employing ELISA, Mitochondrial Viability Staining and the JC-1-Mitochondrial Membrane Potential Assay Kit. The relationship between circ_0030235 and miR-526b was analyzed by dual luciferase reporter assay. The expression of key proteins was analyzed by western blot.

**Results:**

Circ_0030235 was highly expressed in OGD/R-induced H9c2 cells. OGD/R inducement cell viability, while accelerated apoptosis. Besides, the level ROS, cell injury-related factors, mitochondrial membrane potential were notably elevated by OGD/R inducement, while mitochondrial viability was remarkably declined. Whereas, these impacts were all noticeably remitted by circ_0030235 knockdown. miR-526b was a target of circ_0030235. Circ_0030235 knockdown-induced impacts were all notably abrogated by miR-526b inhibition, including the activating impacts on PI3K/AKT and MEK/ERK pathways.

**Conclusions:**

This research implied that circ_0030235 knockdown might remit OGD/R-induced impacts via activation of PI3K/AKT and MEK/ERK pathways and regulation of miR-526b.

## Introduction

Acute myocardial infarction (AMI) is the common clinical manifestation of coronary heart disease (Thiele et al., 2019). AMI can induce mass mortality of cardiomyocytes owing to ischemic injury, followed by the formation of fibrosis scarring that lacks normal electrical conduction capacity, and insufficient blood supply near the infarcted myocardium (Wang et al., 2018). Although great improvements in AMI treatment strategies have been achieved in recent years, the treatment outcomes of patients with heart failure remain unsatisfied (Thum, 2012). Therefore, further understanding of the underlying regulating mechanism is still in need.

Circular RNAs (circRNAs), a set of non-coding RNAs, are characterized by the covalently closed structure which was generated through back splicing (Chen & Yang, 2015; Hentze & Preiss, 2013). Previous investigations discovered that circRNAs were widely expressed in various cell types and dysregulation of circRNAs were found to be associated with cardiovascular diseases, such as atherosclerosis (Holdt et al., 2016), myocardial infarction (MI) (Geng et al., 2016) and heart failure (Devaux et al., 2017). For instance, circRNA_081881 was distinctly down-regulated in the blood samples of MI patients (Yang et al., 2017). Besides, circRNA CAMK2D expression was also discovered to be downregulated in dilated and hypertrophic cardiomyopathy (Khan et al., 2016). Circ_0030235 has been studied as an unfavorable prognostic role for pancreatic ductal adenocarcinoma (Xu et al., 2019). Additionally, RNA-based expression assays of our previous research have been developed to elucidate abnormal circRNA expression in MI cell model, showing some evidences of circ_0030235 upregulation in cardiac tissues of MI patients. This finding has led to interests in understanding the role of circ_0030235 in MI.

Previous researches discovered that circRNAs have conserved microRNA (miRNA) binding sites which make it possible to function as miRNA sponges to regulate the expression of its downstream genes, and thereby exert a role in the pathological and physiological processes (Hall et al., 2019; Wang et al., 2016). It was noted that circRNA CDR1AS could aggravate MI-mediated cardiomyocyte loss through interaction with miR-7a and miR-7a inhibited cardiac infarct size by downregulating downstream target SP1 (Geng et al., 2016). Besides, heart-related circRNA HRCR was discovered to perform protective roles in heart failure via function as a sponge of miR-223, whereas miR-223 enhanced the chances for cardiac hypertrophy (Wang et al., 2016). In additional, miR-433 was involved in the regulation of circNFIB in the progression of cardiac fibrosis (Zhu et al., 2019). These studies indicated that miRNAs have direct involvement in cardiac disease and were known to play important roles in development and treatment of MI (Goretti, Wagner & Devaux, 2014; Mirzavi et al., 2019). MiR-526b-3p was previously reported to promote doxorubicin-evoked cardiotoxicity through modulating STAT3/VEGFA axis (Zhang, Liu & Li, 2020). The aim of this investigation is to test the hypothesis that circ_0030235 might act as a sponge of miR-526b to regulate cardiomyocyte injury in MI.

MEK/ERK signaling pathway exhibited an activation of STAT3 expression which is a target of miR-526b in myocardial ischemia/reperfusion (I/R) (Jin et al., 2018), indicating that its involvement in cardiomyocyte injury. Studies have shown that the MEK/ERK pathway played an important role in the regulation of H9c2 cell inflammatory injury (Shao et al., 2020; Zhu et al., 2020). In addition, reports have previously shown a correlation between PI3K/AKT pathway and miRNA-mediated cardiomyocyte apoptosis (Huang et al., 2020; Xing et al., 2020). In this work, we investigated the regulatory mechanism of circ_0030235 in an MI cell model by treating H9c2 cells with oxygen glucose deprivation/reperfusion (OGD/R). Furthermore, we studied whether its mechanism of cellular inflammatory injury was related to MEK/ERK and PI3K/AKT pathways. This investigation might contribute to the discovery of therapeutic targets of MI.

## Materials and Methods

### Cell culture

H9c2 cells, a rat cardiomyocyte cell line, were obtained from the American Type Culture Collection (ATCC; Manassas, VA, US) and grown in a mixture of Dulbecco’s modified Eagle medium (DMEM; ATCC) containing 10% fetal bovine serum (FBS; ATCC) in a circumstance of 5% CO_2_ at 37 °C. Replacement of the culture medium was conducted every other day.

For OGD/R treatment, H9c2 cells were pre-maintained with DMEM (no glucose) (Gibco, Rockville, MD, USA) in an incubator with a mixture of 0.1% O_2_, 94.9% N_2_, and 5% CO_2_ (OGD treament). 6 h later, cells were then cultivated with DMEM in condition of 95% air and 5% CO_2_ for another 12 h (re-oxygenation treatment). The cells grown in DMEM medium containing 10% FBS under condition of 95% air and 5% CO_2_ were used as control.

### Cell transfection

Short hairpin (sh) RNA targeting circ_0030235 (sh-circ) and its corresponding control (sh-NC) was synthesized and inserted into vectors by GenePharma (Shanghai, China). MiR-526b inhibitor and its corresponding control (miR-NC) were purchased from GenePharma. A transient transfection was performed using Lipofectamine 3000 (Thermo Fisher Scientific, Waltham, MA, USA). In brief, 48 h prior to transfection, 5  × 10^5^ H9c2 cells were plated in a 6-well plate. Cells were transfected with 1 µg shRNA vectors or 0.1 µmol miR inhibitor. After 48 h, cells were subjected to total RNA extraction for qRT-PCR detection.

### Cell viability

After cell transfection and OGD/R treatment, H9c2 cells were seeded into 96-well plates (1  × 10^5^ cells per well) and cultured in a 5% CO_2_ incubator at 37 °C. After that, 10 µL of CCK-8 reagent (GLPBIO) was added to each well, and maintained in the same incubator for 1 h. Thereafter, a microplate reader (BMG Labtech, Oefenburg, Germany) was used for measurement of the absorbance at 450 nm.

### Apoptosis assessment

H9c2 cells were subjected to OGD/R treatment and then transfected with shRNA or miRNA inhibitor. Internucleosomal DNA fragmentation was detected using TUNEL Assay Kit (ab66108; Abcam, Cambridge, UK) to assess cell apoptotic potential. In brief, the adherent cultured H9c2 cells were fixed with 1% (w/v) paraformaldehyde on ice for 15 min. Next, cells were washed in 5 mL of 1 × PBS (pH 7.2−7.4, 0.01 M; Solarbio, Beijing, China) and pelleted by centrifugation. 5 mL of ice-cold 70% (v/v) ethanol was utilized for 30 min of cell incubation. The fixed cells were centrifuged (300 × g) for 5 min to remove the ethanol and re-suspend with 1 mL of Wash Buffer. Then, staining Solution was prepared as follows: 10 µL reaction buffer, 0.75 µL TdT Enzyme, 8 µL FITC-dUTP and 32.25 µL ddH_2_O. Cells were incubated in the indicated Staining Solution for 60 min at 37 °C. Subsequently, cell pellet were re-suspended in 0.5 mL of Propidium Iodide/RNase A Solution and incubated in the dark for 30 min at room temperature. Cell nucleus was stained with DAPI staining solution (10 mM, ab228549; Abcam) for 5 min. For quantification of apoptosis, the cells were observed by a confocal microscope (DMi8, Leica, Wetzlar, Germany) (Ex/Em = 495/519nm, apoptotic cells showed green staining). Images were processed with ImageJ software (Schneider, Rasband & Eliceiri, 2012).

### ROS injury assessment

Intracellular ROS level was assessed by employing the ROS Detection Assay Kit (Catalog #K936, BioVision, San Francisco, CA, USA). Briefly, after OGD/R treatment and cell transfection, cells seeded in 96-well plates (2.5 × 10^4^ cells per well) were allowed to adhere and grow to 70–80% confluency. Next day, the adherent cultured H9c2 cells were added with 100 µL of ROS Label Solution (final concentration 1 ×) diluted in ROS Assay Buffer, re-suspended at 1.5 × 10^6^ cells/mL and incubate for 45 min at 37 °C in the dark. Thereafter, the ROS label was discarded, and then 100 µL of ROS Assay Buffer was added for fluorescence measurement. The fluorescence was immediately detected by using a microplate reader (BMG Labtech) at Ex/Em = 495/529 nm.

### Cellular injury assessment

After OGD/R inducement and cell transfection, the productions of creatine kinase-MB (CK-MB) and cardiac troponin I (cTnI) were measured using CK-MB ELISA Kit (Catalog #E4608-100, BioVision) and Rat cTnI ELISA Kit (ab246529, Abcam). Besides, measurement of mitochondria viability was conducted by using Mitochondrial Viability Staining (Catalog #K239-100; BioVision). Furthermore, Mitochondrial membrane potential was measured by using JC-1-Mitochondrial Membrane Potential Assay Kit (a final concentration of 10 µg/mL, Catalog #1130-5; BioVision).

### Dual luciferase reporter assay

Constructions of the recombined overexpression vectors wild-type (wt) circ_0030235 and mutant type (mut) of circ_0030235 were completed by GenePharma. Afterwards, co-transfections of circ_0030235-wt/circ_0030235-mut and miR-526b mimic/miR-NC were completed by using Lipofectamine 3000 (Thermo Fisher Scientific). After transfected for 48 h, the luciferase activities of these transfected cells were determined by employing the Luciferase Assay Kit (Promega, Madison, WI, USA).

### Quantitative reverse transcription polymerase chain reaction (qRT-PCR)

Trizol reagent (TAKARA, Beijing, China) was used for the isolation of total RNAs with the existence of DNase I (Sangon Biotech, Shanghai, China). The Nanodrop 2000 system (Thermo Fisher Scientific) was employed for measurement of the concentration of isolated RNAs. Afterwards, synthesis of the first strand of cDNA was completed with the help of PrimeScript RT Master Mix (TAKARA). After that, qRT-PCR was carried out by using SYBR Premix Ex Taq™ (TAKARA). The primers used were as follows: circ_0030235, 5′-AAT TTG ACA ACC CGG ACA CT-3′ (forward primer) and 5′-CAG ACG TGT GTG TGG AGT GA-3′ (reverse primer); miR-526b, 5′-GCG ACT CTT GAG GGA AGC ACT-3′ (forward primer) and 5′-AGT GCA GGG TCC GAG GTA TT-3′; GAPDH, 5′-AAG GAA ATG AAT GGG CAG CC-3′ (forward primer) and 5′-TAG GAA AAG CAT CAC CCG GA-3′ (reverse primer); U6, 5′-CTC GCT TCG GCA GCA CAT-3′ (forward primer) and 5′-TTT GCG TGT CAT CCT TGC G-3′ (reverse primer). The internal references for miR-526b and circ_0030235 were U6 and GAPDH, respectively. Gene expression was calculated by utilizing the 2^−^^△△^^Ct^ method.

### Western blot

Cellular protein extraction was conducted by using RIPA Lysis Buffer (BosterBio, California, USA) with the presence of Protease inhibitor (Sangon Biotech). BCA method (Solarbio) was employed for protein concentration determination. After completing sodium dodecyl sulfate polyacrylamide gel electrophoresis (SDS-PAGE), the proteins were transferred onto the nitrocellulose membrane. After that, 5% bovine serum albumin (BSA; AG Scientific, Inc., San Diego, CA, USA) blocking was carried out on the membranes at room temperature for 2 h. Thereafter, primary antibody incubation was performed on the membranes at 4 °C for 16 h. After rinsing, the HRP-marked secondary antibody (ab205718; Abcam) incubation was conducted at room temperature for 1 h. After finishing that, an ECL reagent was added to cover the membranes and the signals were then detected by employing LAS-3000 system (FUJIFILM, Tokyo, Japan). QuantityOne software was used to analyze the intensity of the protein bands obtained from western blot.

The primary antibodies used in this research were detailed below: Bcl-2 (1:1000, #3033-100, BioVision), Bax (1:2000, #3032-100, BioVision), cleaved-caspase-3 (1:2000, #orb126608; Biorbyt, Cambridge, UK), cleaved-caspase-9 (1:1000, #orb227889, Biorbyt), cytochrome c (1:2000, #3025-100; BioVision), p-PI3K (1:800, #orb14998; Biorbyt), t-PI3K (1:2000, #orb137259, Biorbyt), p-AKT (1:2000, #3257-100, BioVision), t-AKT (1:1000, #A1117-50; BioVision) and *β*-actin (1:5000, #ab119716; Abcam).

### Statistical analysis

The data were expressed as the mean ± standard deviation (S.D.) from three independent experiments. Data analysis was performed by employing SPSS 17.0 software (SPSS Inc, Armonk, USA). One-way analysis of variance (ANOVA) and Student’s *t*-test were respectively employed for comparison among multi-groups and between two groups. *P* <  0.05 was acknowledged to be statistically significant.

## Results

### Circ_0030235 was up-regulated in OGD/R-induced H9c2 cells

**Figure 1:**
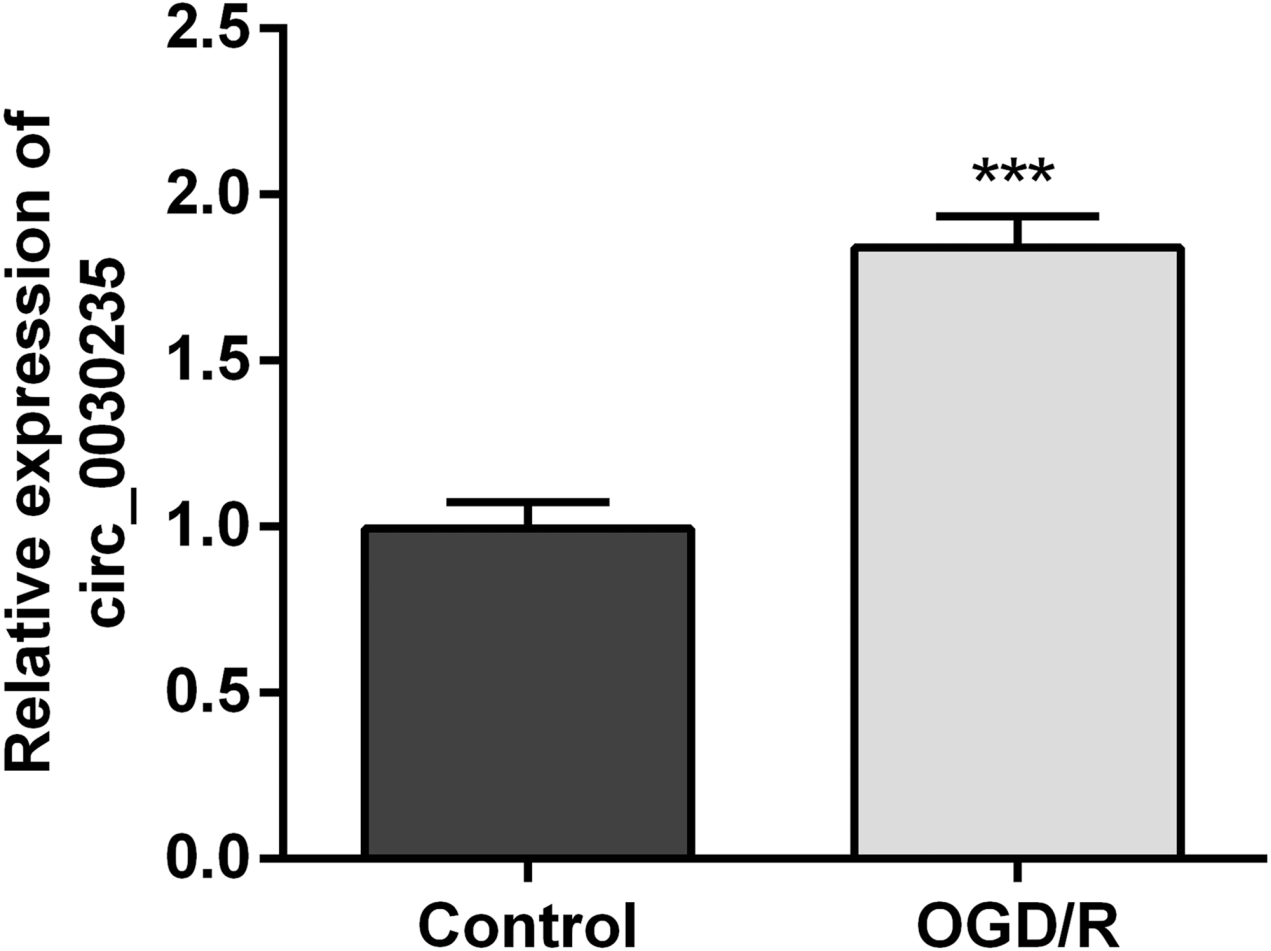
Circ_0030235 was up-regulated in OGD/R-treated H9c2 cells. H9c2 cells were subjected to OGD (6 h) and then cultured in normoxic conditions for 12 h. qRT-PCR showing the effect of OGD/R treatment on Circ_0030235 upregulation in H9c2 cells. The data were expressed as the mean ± S.D. from three independent experiments. ^∗∗∗^*P* < 0.001.

The previous investigation clarified that circ_0030235 exerted carcinogenic impacts on pancreatic ductal adenocarcinoma (Xu et al., 2019). However, its functions in MI remain unclear. Therefore, we measured its expression in OGD/R-induced H9c2 cells. Results displayed that circ_0030235 expression was noticeably up-graded by OGD/R inducement (*P* <  0.001, Fig. 1), which implied that circ_0030235 expression might be associated with the development of MI.

### Circ_0030235 silencing alleviated the decreased cell viability and cell apoptosis caused by OGD/R

**Figure 2:**
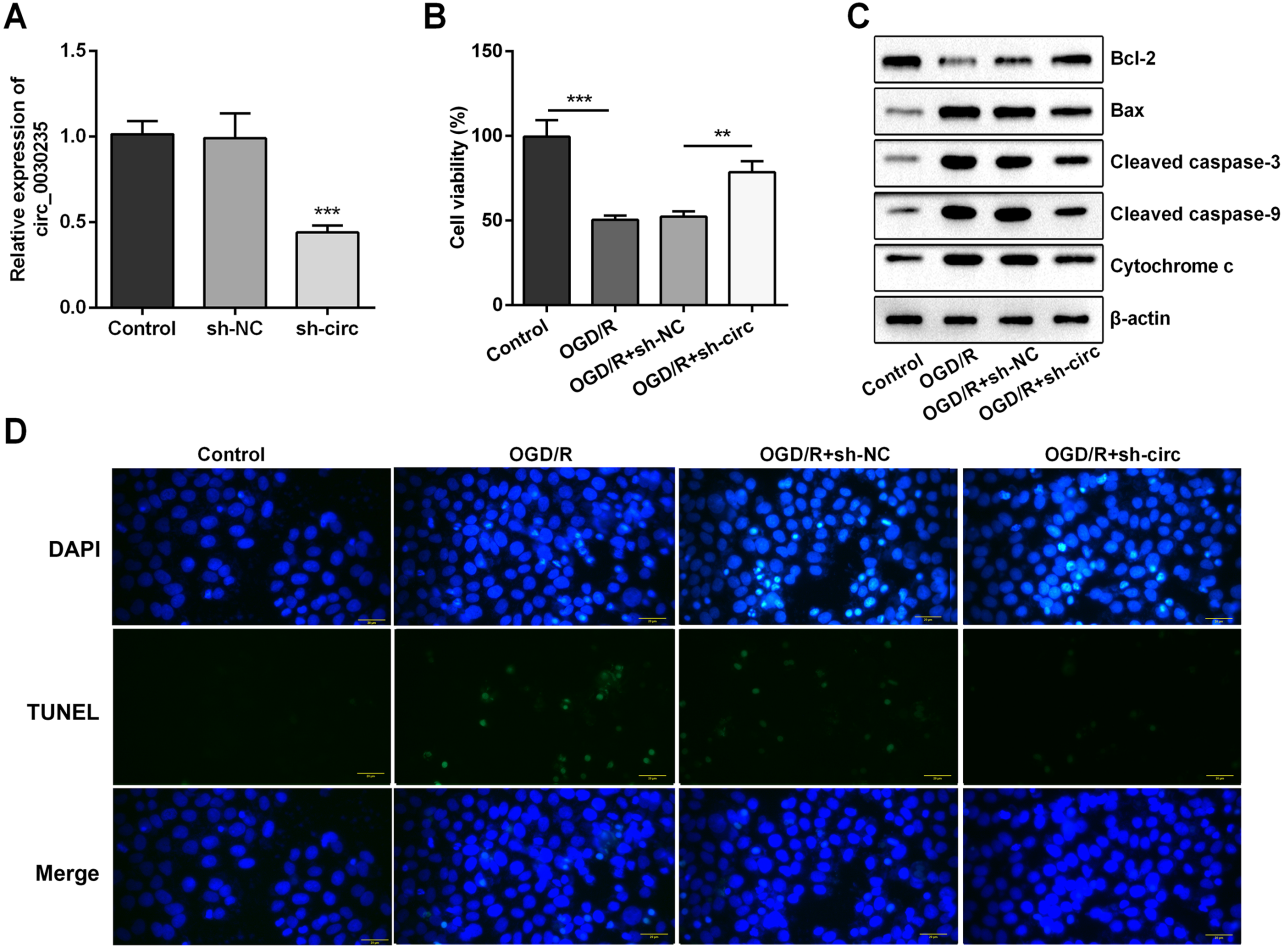
Knockdown of Circ_0030235 remitted OGD/R-decreased cell viability and -induced apoptosis. H9c2 cells were transfected with sh-circ and sh-NC for 48 h, followed by 6 h of OGD exposure and 12 h of re-oxygenation treatment. (A) qRT-PCR showing the effective knockdown of sh-circ on Circ_0030235 expression. (B) CCK-8 assay showed that the viability of OGD/R-treated cells was increased by sh-circ transfection. (C) Western blot analysis detecting the levels of Bcl-2, Bax, Cleaved caspase-3, Cleaved caspase-9 and Cytochrome c showed that the expression of pro-apoptotic protein was decreased by sh-circ transfection in OGD/R-treated cells. (D) Representative scanning picture of TUNEL showing decreased apoptosis of OGD/R-treated and sh-circ-transfected H9c2 cells. Scale bar = 20 µm. The data were expressed as the mean ± S.D. from three independent experiments. ^∗∗^*P* < 0.01, ^∗∗∗^*P* < 0.001.

For determining the efficacy of circ_0030235 expression on the characteristics of OGD/R-induced H9c2 cells, knockdown of circ_0030235 was performed on H9c2 cells. Results showed that circ_0030235 expression was declined by over two-fold after sh-circ transfection (*P* < 0.001, Fig. 2A), which meant the transfection efficiency was high. Besides, the CCK-8 assay showed that the viability of H9c2 cells was distinctly repressed after OGD/R inducing (*P* < 0.001, Fig. 2B). Apoptosis was observed in attenuated Bcl-2, augmented Bax, cleaved caspase-3, cleaved caspase-9, cytochrome c and increased TUNEL staining cells (Figs. 2C and 2D). However, the following results verified that the aforementioned impacts triggered by OGD/R inducement were all distinctly diminished by circ_0030235 knockdown (*P* < 0.01 or *P* < 0.001, Figs. 2B–2D). Combining the aforementioned results led to a conclusion demonstrating that knockdown of circ_0030235 notably remitted the decreased cell viability and elevated apoptosis on H9c2 cells induced by OGD/R.

### Knockdown of circ_0030235 remitted OGD/R-induced H9c2 cell injury

**Figure 3:**
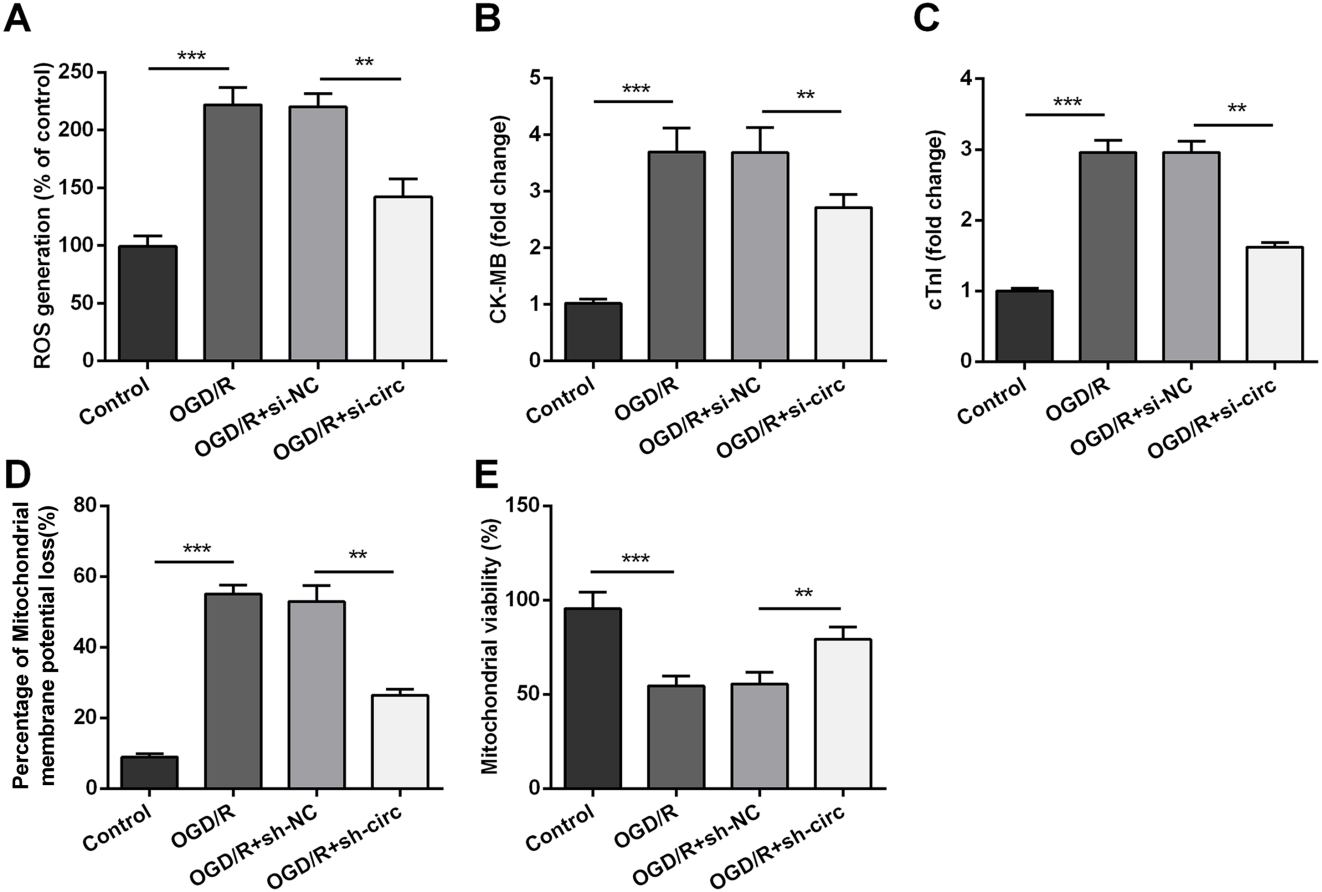
Knockdown of Circ_0030235 remitted OGD/R-induced ROS and mitochondrial injury. H9c2 cells were transfected with sh-circ and sh-NC for 48 h, followed by 6 h of OGD exposure and 12 h of re-oxygenation treatment. (A) Measurement of ROS generation using ROS Detection Assay Kit showed that OGD/R+sh-circ group has a lower ROS generation than that in OGD/R+sh-NC group. ELISA assays were utilized to confirm the inhibitory effects of sh-circ transfection on the productions of (B) CK-MB and (C) cTnI in OGD/R-treated cells. (D) JC-1-Mitochondrial Membrane Potential assay showed that silencing Circ_0030235 reversed the enhancement of mitochondrial membrane potential loss triggered by OGD/R treatment. (E) Mitochondrial Viability Staining revealed that silencing Circ_0030235 enhanced the mitochondrial viability of OGD/R-treated cells. The data were expressed as the mean ± S.D. from three independent experiments. ^∗∗^*P* < 0.01, ^∗∗∗^*P* < 0.001.

For determining the influences of circ_0030235 knockdown on OGD/R-induced H9c2 cell injury, the generation of ROS was measured. The result displayed that the OGD/R inducement noticeably up-graded ROS generation (*P* < 0.001, Fig. 3A). Moreover, productions of the MI biomarkers CK-MB and cTnI were detected. It came out that the productions of CK-MB and cTnI were both noticeably augmented after OGD/R inducement (both *P* <0.001, Figs. 3B and 3C). Besides, OGD/R inducement also induced Mitochondrial membrane potential loss (*P* < 0.001, Fig. 3D), and Mitochondrial viability reduction (*P* < 0.001, Fig. 3E). Whereas, results of subsequent experiments suggested that these OGD/R-induced impacts were all distinctly reversed by knockdown of circ_0030235 (all *P* < 0.01, Figs. 3A–3E). These results indicated that OGD/R-induced cell injury could be noticeably remitted by circ_0030235 knockdown.

### miR-526b was targeted and regulated by circ_0030235

**Figure 4:**
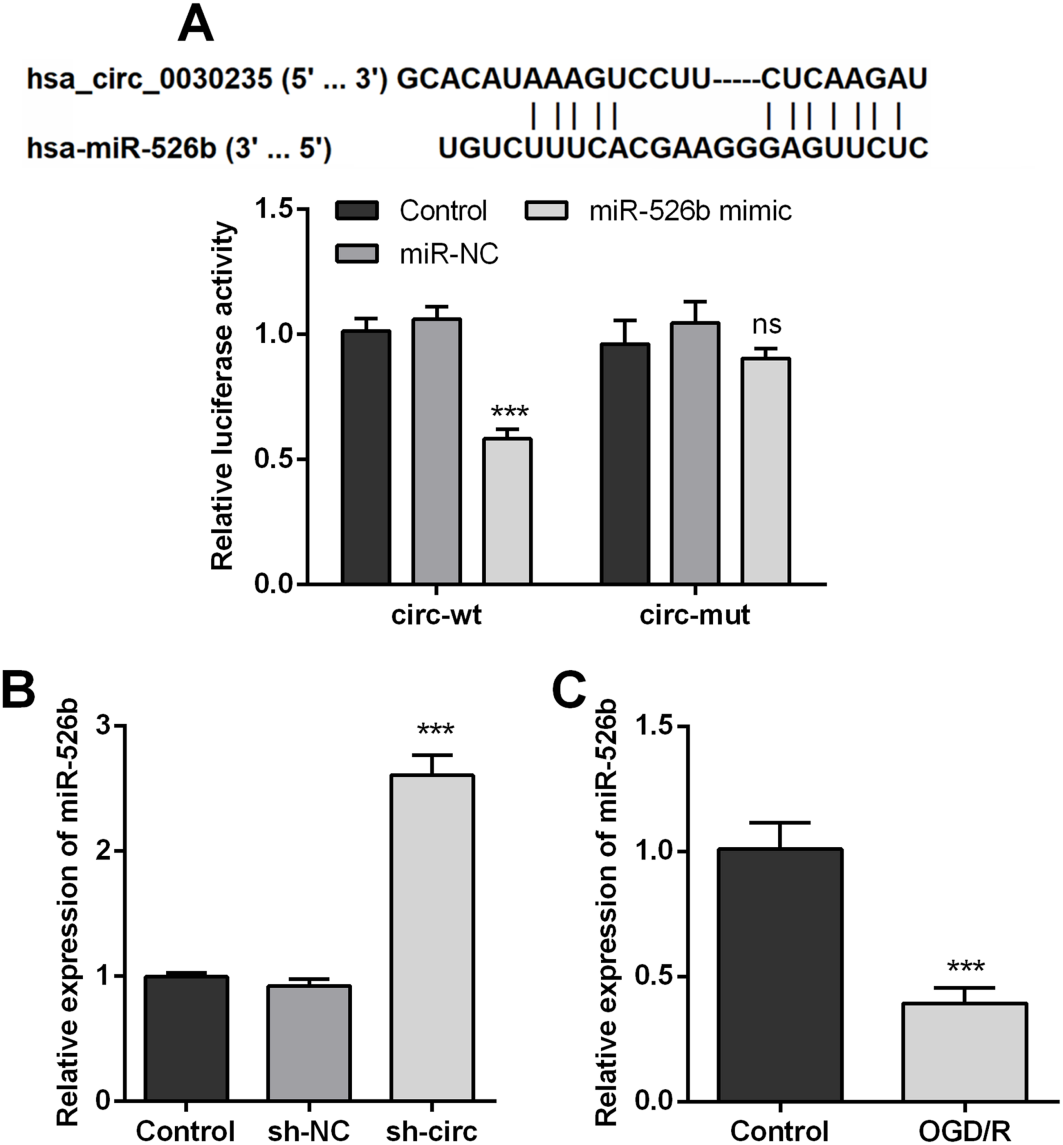
MiR-526b was targeted and regulated by Circ_0030235. (A) Upper panel: schematic diagram depicting complementary sequences of miR-526b recognized by Circ_0030235; lower panel: luciferase reporter assay showed that co-transfection of miR-526b mimic with the constructed Circ_0030235-wt plasmid obviously decreased luciferase activity. (B) Silencing Circ_0030235 enhanced the expression of miR-526b in H9c2 cells, as evidenced by qRT-PCR assay. (C) H9c2 cells were subjected to OGD (6 h) and then cultured in normoxic conditions for 12 h. qRT-PCR showing the decreased miR-526b expression levels in OGD/R group. The data were expressed as the mean ±S.D. from three independent experiments. ^∗∗∗^*P* < 0.001, ns, not significant.

In order to disclose how circ_0030235 worked in remitting OGD/R-induced impacts, the Circular RNA Interactome database was used for the analysis of the downstream gene of circ_0030235. The results displayed that miR-526b was predicted to be a target gene of circ_0030235 and the corresponding targeting sequence was shown in Fig. 4A. This result was verified by the outcomes of dual luciferase reporter assay. The luciferase activity was distinctly declined after the co-transfection of circ_0030235-wt and miR-526b mimic (*P* < 0.001). Based on this result, miR-526b expression in circ_0030235 knockdown and OGD/R-induced H9c2 cells were respectively measured. Outcomes displayed that miR-526b expression was distinctly augmented by circ_0030235 knockdown (*P* < 0.001, Fig. 4B), while was remarkably declined by OGD/R inducement (*P* <  0.001, Fig. 4C). These results manifested that miR-526b was negatively regulated by circ_0030235.

### Knockdown of circ_0030235 remitted OGD/R-decreased cell viability and -induced apoptosis via modulating miR-526b expression

**Figure 5:**
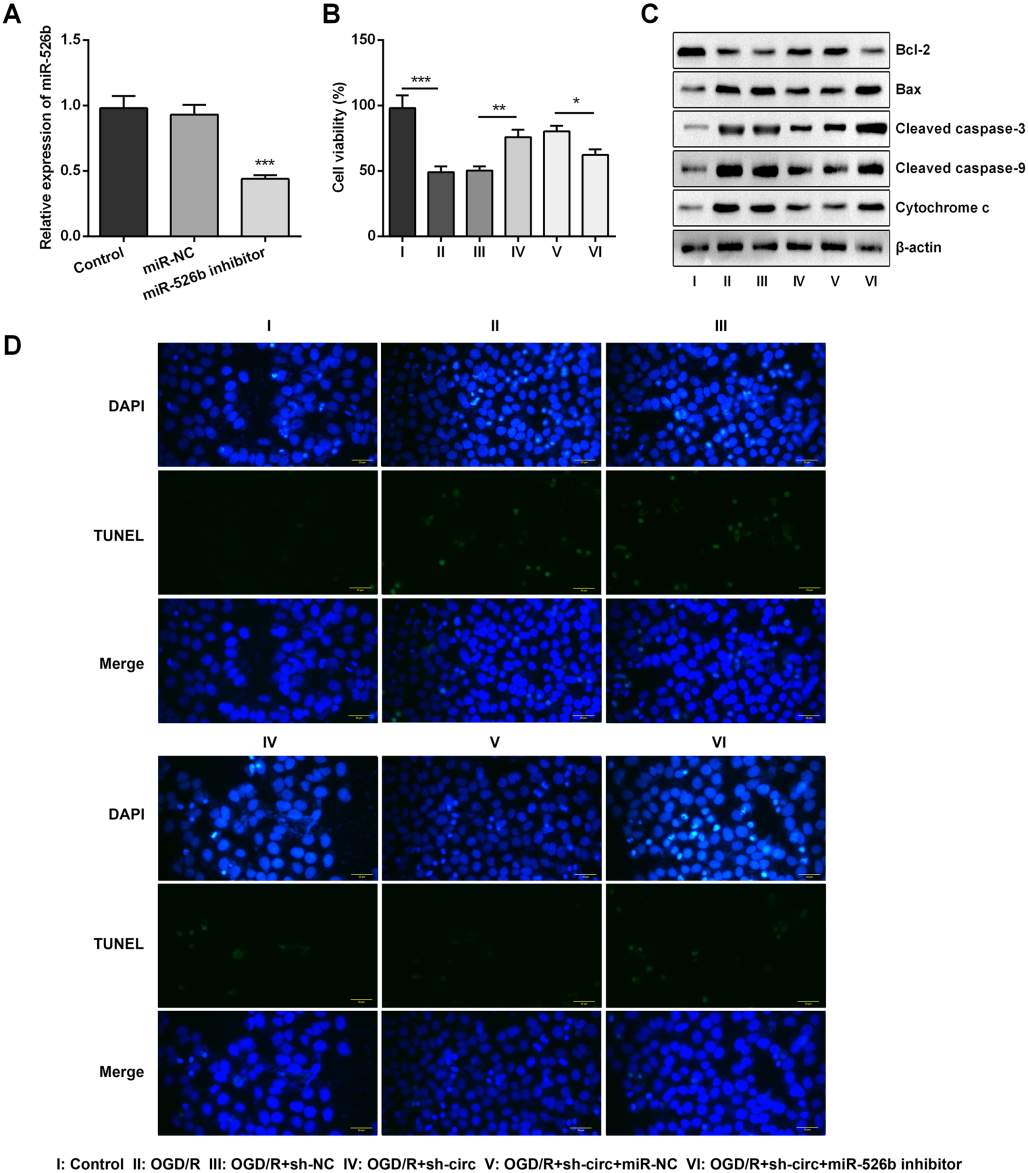
Knockdown of Circ_0030235 remitted OGD/R-decreased cell viability and -induced apoptosis via modulating miR-526b expression. H9c2 cells were co-transfected with sh-circ/sh-NC and miR-526b inhibitor/miR-NC for 48 h, followed by 6 h of OGD exposure and 12 h of re-oxygenation treatment. (A) qRT-PCR showing the effective knockdown of miR-526b inhibitor on miR-526b expression. (B) CCK-8 assay showed that the viability of OGD/R-treated cells was decreased by co-transfection of sh-circ and miR-526b inhibitor. (C) Western blot analysis detecting the levels of Bcl-2, Bax, Cleaved caspase-3, Cleaved caspase-9 and Cytochrome c showed that the expression of pro-apoptotic protein was increased by co-transfection of sh-circ and miR-526b inhibitor in OGD/R-treated cells. (D) Representative scanning picture of TUNEL showing miR-526b knockdown reversed the inhibitory effect of sh-circ transfection on apoptosis in OGD/R-treated H9c2 cells. Scale bar = 20 µm. The data were expressed as the mean ±S.D. from three independent experiments. ^∗^*P* < 0.05, ^∗∗^*P* < 0.01, ^∗∗∗^*P* < 0.001.

Based on the results of Figs. 4A–4C, miR-526b inhibition was conducted for disclosing the regulatory mechanism of circ_0030235 knockdown. Data shown in Fig. 5A suggested that miR-526b expression was repressed twofold after transfection of miR-526b inhibitor (*P* < 0.001). Furthermore, the following outcomes demonstrated that miR-526b inhibition noticeably abrogated circ_0030235 knockdown-triggered impacts on cell viability and apoptosis (*P* < 0.05, Figs. 5B–5D). Collectively, these results implied that the knockdown of circ_0030235 could have functioned via regulating miR-526b.

### Knockdown of circ_0030235 remitted OGD/R-induced H9c2 cell injury via regulating miR-526b

**Figure 6:**
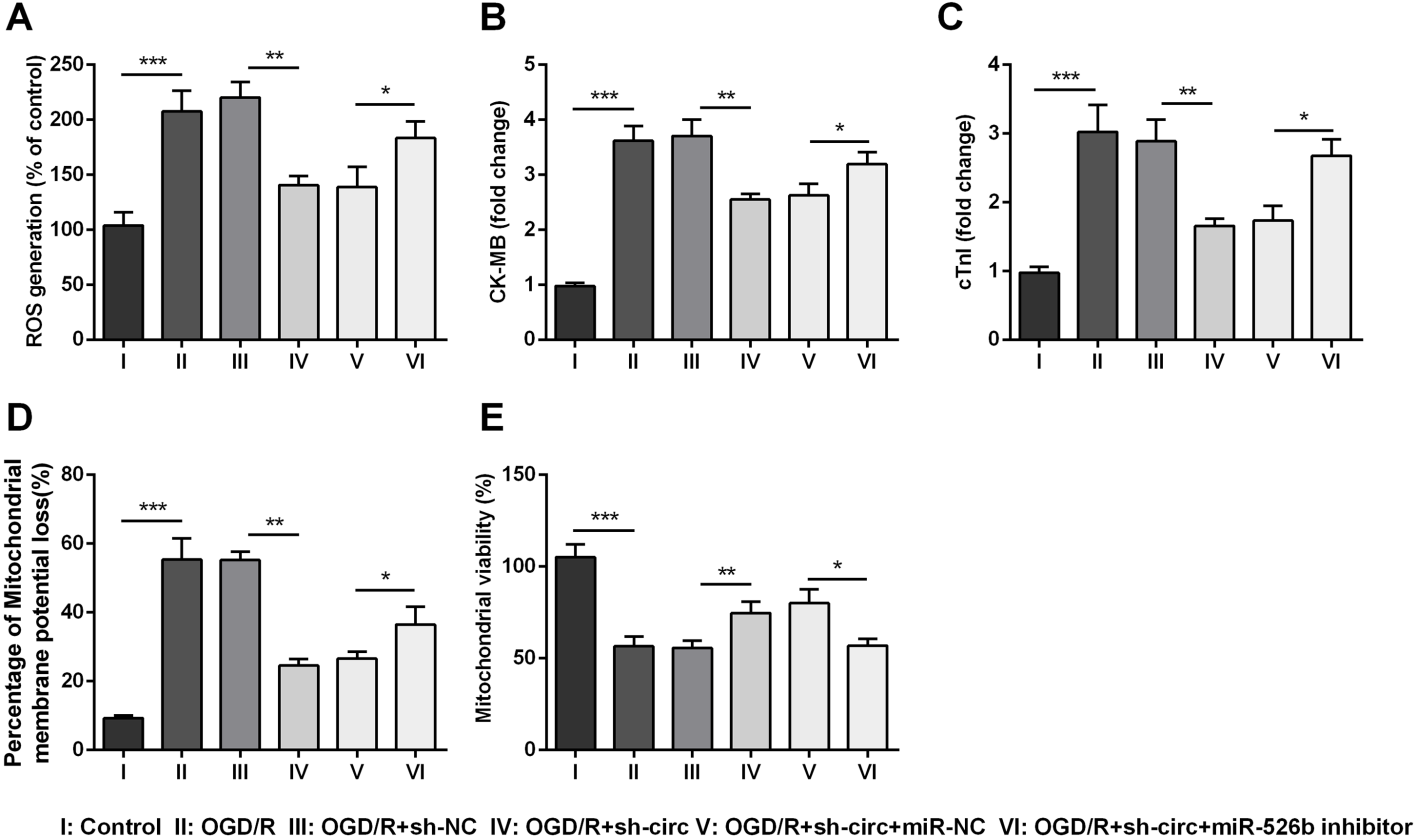
Knockdown of Circ_0030235 remitted OGD/R-induced H9c2 cell injury via regulating miR-526b. H9c2 cells were co-transfected with sh-circ/sh-NC and miR-526b inhibitor/miR-NC for 48 h, followed by 6 h of OGD exposure and 12 h of re-oxygenation treatment. (A) Measurement of ROS generation using ROS Detection Assay Kit showed that OGD/R+sh-circ+miR-526b inhibitor group has a higher ROS generation than that in OGD/R+sh-circ+miR-NC group. ELISA assays were utilized to confirm the enhancement of (B) CK-MB and (C) cTnI levels in OGD/R+sh-circ+miR-526b inhibitor group. (D) JC-1-Mitochondrial Membrane Potential assay showed that miR-526b knockdown reversed the reduction of mitochondrial membrane potential loss triggered by sh-circ in OGD/R-treated cells. (E) Mitochondrial Viability Staining revealed that miR-526b knockdown inhibited the mitochondrial viability of OGD/R-treated and sh-circ-transfected cells. The data were expressed as the mean ±S.D. from three independent experiments. ^∗^*P* < 0.05, ^∗∗^*P* < 0.01, ^∗∗∗^*P* < 0.001.

Additionally, the influences of miR-526b inhibition on productions of ROS, CK-MB, cTnI, mitochondrial membrane potential and mitochondrial viability were also measured. Results showed that circ_0030235 knockdown-evoked impacts on these parameters were all noticeably abrogated by miR-526b inhibition (*P* < 0.05 or *P* < 0.01, Figs. 6A– 6E). Combining with the aforementioned results, a conclusion could be made demonstrating that knockdown of circ_0030235 remitted OGD/R induced cell injury via regulation of miR-526b.

### Knockdown of circ_0030235 activated PI3K/AKT and MEK/ERK pathways via regulation of miR-526b

**Figure 7:**
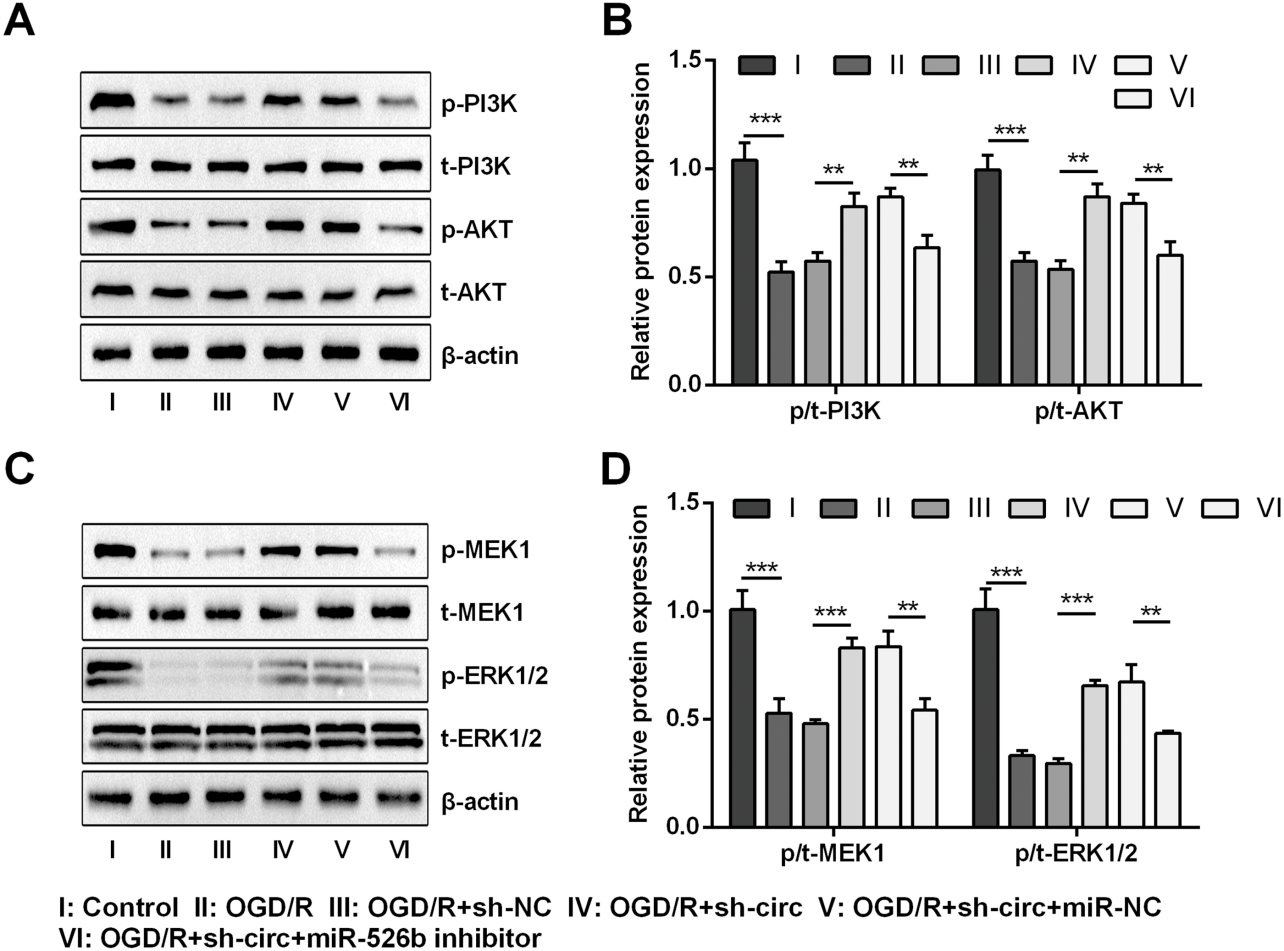
Knockdown of Circ_0030235 activated PI3K/AKT and MEK/ERK pathways via regulation of miR-526b. H9c2 cells were co-transfected with sh-circ/sh-NC and miR-526b inhibitor/miR-NC for 48 h, followed by 6 h of OGD exposure and 12 h of re-oxygenation treatment. (A–B) Western blot analysis showed that silencing Circ_0030235 increased the expression of p-PI3K and p-AKT in OGD/R-treated cells, while miR-526b knockdown partially restrained its effect through ceRNA mechanism. (C–D) Western blot analysis showed that silencing Circ_0030235 increased the expression of p-MEK1 and p-ERK1/2 in OGD/R-treated cells, while miR-526b knockdown partially restrained its effect through ceRNA mechanism. The data were expressed as the mean ±S.D. from three independent experiments. ^∗∗^*P* < 0.01, ^∗∗∗^*P* < 0.001.

For uncovering the underlying mechanism of circ_0030235 and miR-526b towards OGD/R-induced H9c2 cells, the levels of pivotal proteins associated with PI3K/AKT and MEK/ERK pathways were measured. Results exhibited that the ratios of p/t-PI3K and p/t-AKT that participated in the PI3K/AKT pathway were both distinctly declined by OGD/R inducement (both *P* <  0.001, Figs. 7A and 7B). Likewise, the ratios of p/t-MEK1 and p/t-ERK1/2 were also noticeably reduced after OGD/R inducement (both *P* <  0.001, Figs. 7C and 7D). However, the loss-of-function studies demonstrated that these impacts were all observably remitted by circ_0030235 knockdown (*P* < 0.01 or *P* < 0.001, Figs. 7A–7D). Whereas, circ_0030235 knockdown-triggered impacts were then notably offset by miR-526b inhibition (all *P* < 0.01, Figs. 7A–7D). Combining the aforementioned results led to a conclusion demonstrating that circ_0030235 knockdown could reversed OGD/R-induced inactivation in PI3K/AKT and MEK/ERK pathways through up-regulating miR-526b.

## Discussion

In our research, we preliminarily investigated the regulating mechanism of circ_0030235 on OGD/R-induced H9c2 cells. Results demonstrated that circ_0030235 expression was remarkably enhanced by OGD/R inducement. Besides, knockdown of circ_0030235 noticeably relived OGD/R-evoked inhibitory impacts on the viability of H9c2 cells, and the facilitating effects on the apoptosis, ROS injury and expression of apoptosis and cell injury-related factors. miR-526b was a target of circ_0030235 and miR-526b inhibition noticeably diminished knockdown of circ_0030235-triggered impacts. These data implied that knockdown of circ_0030235 could have achieved its protective role in OGD/R-evoked H9c2 cells through the regulation of miR-526b.

MI is a crucial clinical challenge and explaining in detail its molecular events would help to highlight our strategies for reducing the death rate in patients with cardiovascular diseases (Chatterjee & Levy, 2020; Pollard, 2000). Thus, exploring the molecular regulator of cardiomyocytes activity will introduce a new insight into MI treatment. Our study utilized *in vitro* study, which provided some important results into the mechanism regulation of circRNA in MI. Cardiomyocytes, isolated from rat, were usually utilized for investigation of molecular alternation underlying MI. Due to a disadvantage of mass animals usage, primary cardiomyocytes cannot provide a comprehensive basis for experimental studies (Watkins, Borthwick & Arthur, 2011). The H9c2 cells derived from rat embryonic heart were characterized by heart-specific morphological structures (Hescheler et al., 1991) and myocardial cell specific markers (Branco et al., 2015). H9c2 cells are currently used *in vitro* as a simulacrum for myocardial researches (Ge et al., 2019; Zhou, Richards & Wang, 2019).

In recent years, accumulating investigations have disclosed the association between circRNAs and cardiovascular diseases (Devaux et al., 2017; Geng et al., 2016; Holdt et al., 2016). In the present study, RT-qPCR results first indicated that circ_0030235 expression level has been increased in OGD/R-treated H9c2 cells (Fig. 1). Also, data showed that knockdown of circ_0030235 effectively enhanced cell viability and reduced OGD/R-evoked H9c2 cell apoptosis (Fig. 2), consistent to the effects of circHIPK3 in IR-treated cardiomyocytes (Bai et al., 2019). Apoptosis is one of the crucial pathological mechanisms of I/R induced cell damage. A study demonstrated that knockdown of circRNA_101237 remitted anoxia/reoxygenation (A/R)-evoked injury in cardiomyocytes exhibiting as reducing the level of cell apoptosis (Sethi et al., 1991). Bcl-2 was previously validated to possess the capacity of blocking the release of Cytochrome c through keeping the mitochondrial permeability transition pore to be closed. Cytochrome c was once reported to work through interacting with Apaf-1, which finally block apoptosis induction and caspase activation (Azimian et al., 2018; Liu et al., 2018). We found circ_0030235 exerted its activity on regulating myocardial cell apoptosis and was necessary for Bcl-2 and cytochrome c expression under OGD/R treatment (Fig. 2C).

Moreover, our study also demonstrated that silencing circ_0030235 reversed the enhancement of ROS generation, CK-MB and cTnl expression triggered by OGD/R treatment (Fig. 3). ROS generation was noticeably increased in the postischemic myocardium (Lv et al., 2014). Besides, ROS accumulation during the I/R process could effectively accelerate cardiomyocyte apoptosis via the mitochondria apoptosis pathway. Recent studies have shown that multiple circRNAs regulated ROS production and promoted ROS-triggered cell death, apoptosis and inflammatory response (Saaoud et al., 2021). Here, our findings first exposed that OGD/R treatment functions as a positive regulator on increasing the expression of myocardial damage biomarkers (CK-MB and cTnl) (Xu et al., 2020); however, knockdown of circ_0030235 alleviated the process of myocardial damage triggered by OGD/R (Fig. 3). In addition to circ_0030235 investigated in this study, other literatures have discovered several circRNAs that can play important roles in MI. For example, it was shown that knockdown of circROBO2 was able to significantly inhibit the releasing of CK-MB and LDH by enhancing the expression levels of miR-1184 (Chen et al., 2021).

Mounting previous investigation indicated that circRNAs perform their roles in cardiovascular diseases via function as sponges of miRNAs (Goodall & Wickramasinghe, 2021; Kristensen et al., 2019). In this research, combining bioinformatics and qRT-PCR analysis, we primarily speculated that circ_0030235 has a relationship with miR-526b and then performed dual-luciferase reporter assay to verify that circ_0030235 directly sponged to miR-526b (Fig. 4). To further investigate whether circ_0030235 could function by miR-526b in MI, we co-transfected sh-circ and miR-526b inhibitor into H9c2 cells. We found that knockdown of circ_0030235 effectively relieved OGD/R-evoked ROS and mitochondrial injury in H9c2 cells, while these effects were then verified to be notably reversed by miR-526b inhibition (Fig. 5 and Fig. 6). In addition, previous research indicated that up-regulation of circRNA Cdr1as enhanced the apoptosis of mouse cardiac myocyte (MCM) cells through regulating miR-7a (Geng et al., 2016). Another study demonstrated that knockdown of circ_0010729 released OGD-evoked repressing impacts on the growth and migration of HCM cells through increasing miR-145-5p expression (Jin & Chen, 2019). It was proved that silencing circDENND2A effectively depressed the viability and migration, while enhanced apoptosis of H9c2 cells after OGD treatment via modulating the expression of miR-34a (Shao et al., 2020). Moreover, silencing circNCX1 noticeably mitigated MI injury through the regulation of miR-133a-3p (Li et al., 2018). Up-regulation of miR-34a and miR-21 was also recorded to augment the migratory capacity of cardiac fibroblast and cardiac stem cells (Huang et al., 2014; Zhou et al., 2016). Additionally, a previous report demonstrated that the production of ROS was significantly decreased after miR-124 and miR-506 overexpression in H_2_O_2_-treated human cardiomyocytes (Zhang et al., 2017). Coincidentally, our research identified a novel circ_0030235 as a sponge for miR-526b to exert its function in OGD/R-induced H9c2 cell injury.

In recent years, substantial researches have proved the participation of PI3K/AKT and MEK/ERK pathways in the regulation of MI (Ren & Wang, 2018; Thomas et al., 2015). In our investigation, we found that both PI3K/AKT and MEK/ERK pathways were activated during circ_0030235 knockdown in order to relieve OGD/R-evoked H9c2 cell injury, while further research verified that inhibition of miR-526b partially abolished these effects (Fig. 7). In other words, circ_0030235 knockdown might have performed its protective roles in OGD/R-evoked H9c2 cells via up-regulating miR-526b expression, which was consistent with the aforementioned evidence. For instance, a previous report demonstrated that forced expression of miR-384 noticeably repressed I/R-evoked autophagy in H9c2 cells via activation of the PI3K/AKT pathway (Zhang et al., 2019). Similarly, another study demonstrated that miR-496 upregulation notably remedied hypoxia reoxygenation (H/R)-evoked injury, exhibiting as reducing apoptosis and increasing proliferation, via activation of the PI3K/AKT pathway (Jin & Ni, 2020). Besides, it was disclosed that lncRNA GAS5 silence exerted protective efficacy on hypoxia-evoked H9c2 cell injury via activation of MEK/ERK and PI3K/AKT pathways, while miR-142-5p inhibition notably diminished these effect (Du et al., 2019). Additionally, a previous investigation discovered that miR-21 was up-regulated and PTEN/PI3K/AKT pathway was activated in the protective process of gastrodin against hypoxia-evoked H9c2 cell injury (Xing & Li, 2019).

## Conclusions

In summary, our data demonstrated that knockdown of circ_0030235 exhibited myocardial protective effects by increasing cardiomyocyte viability and inhibiting cardiomyocyte apoptosis and alleviating post-MI cell injury in part through miR-526b binding mechanism. In addition, circ_0030235 acts as a miRNA sponge to inhibit miR-526b function and then regulates the PI3K/AKT and MEK/ERK signaling axis in cardiomyocyte. The study highlighted a new connection between circ_0030235 and miR-526b in the construction of a protection for cardiomyocytes from OGD/R injury, which may offer novel insights and therapeutic strategies for MI prevention and treatment.

## Supplemental Information

10.7717/peerj.11482/supp-1 Supplemental Information 1 Western blot Figure

10.7717/peerj.11482/supp-2 Supplemental Information 2 Raw numeric data

## Additional Information and Declarations

### Data Availability

The following information was supplied regarding data availability: Raw data are available as a Supplementary File.
