# Supplementary figures and images for: Circ_0030235 knockdown protects H9c2 cells against OGD/R-induced injury via regulation of miR-526b
Source: PeerJ. 2021 Nov 16;9:e11482. doi: 10.7717/peerj.11482 (PMC8603820; doi:10.7717/peerj.11482)

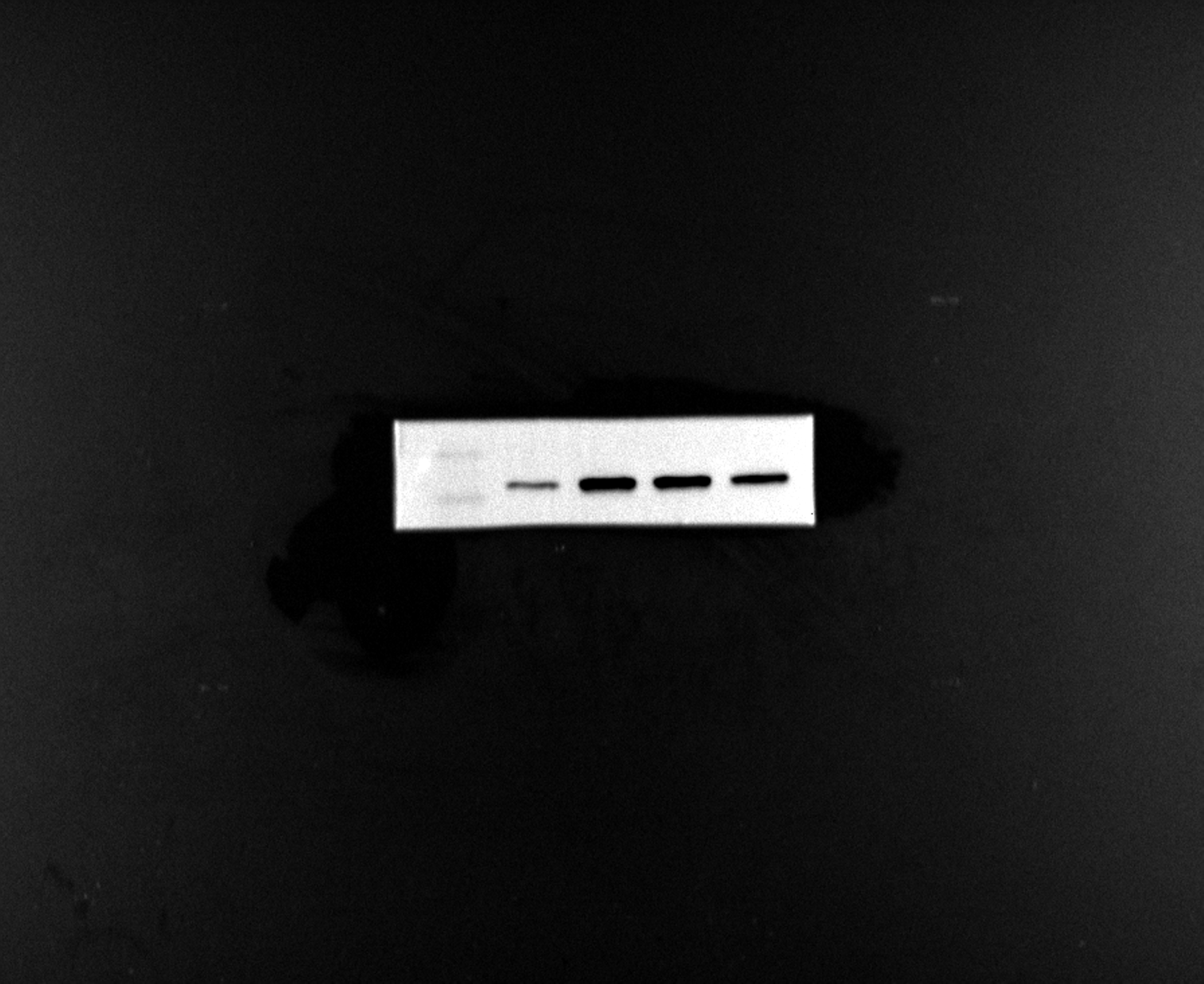

Supplement: Supplemental Information 1 [file peerj-09-11482-s001.zip › Western blot Figure/Fig2c-Bax.Tif]

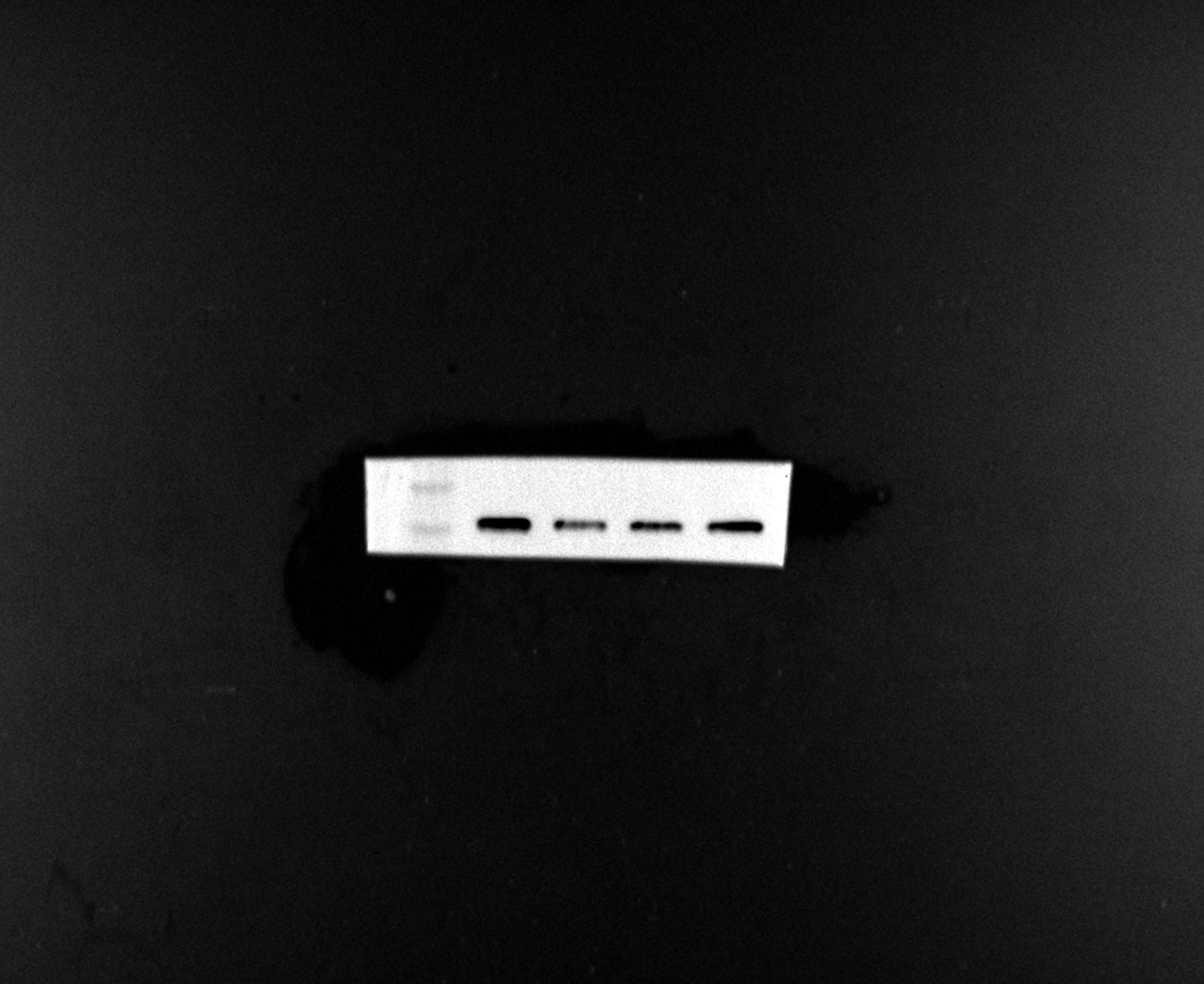

Supplement: Supplemental Information 1 [file peerj-09-11482-s001.zip › Western blot Figure/Fig2c-Bcl-2.Tif]

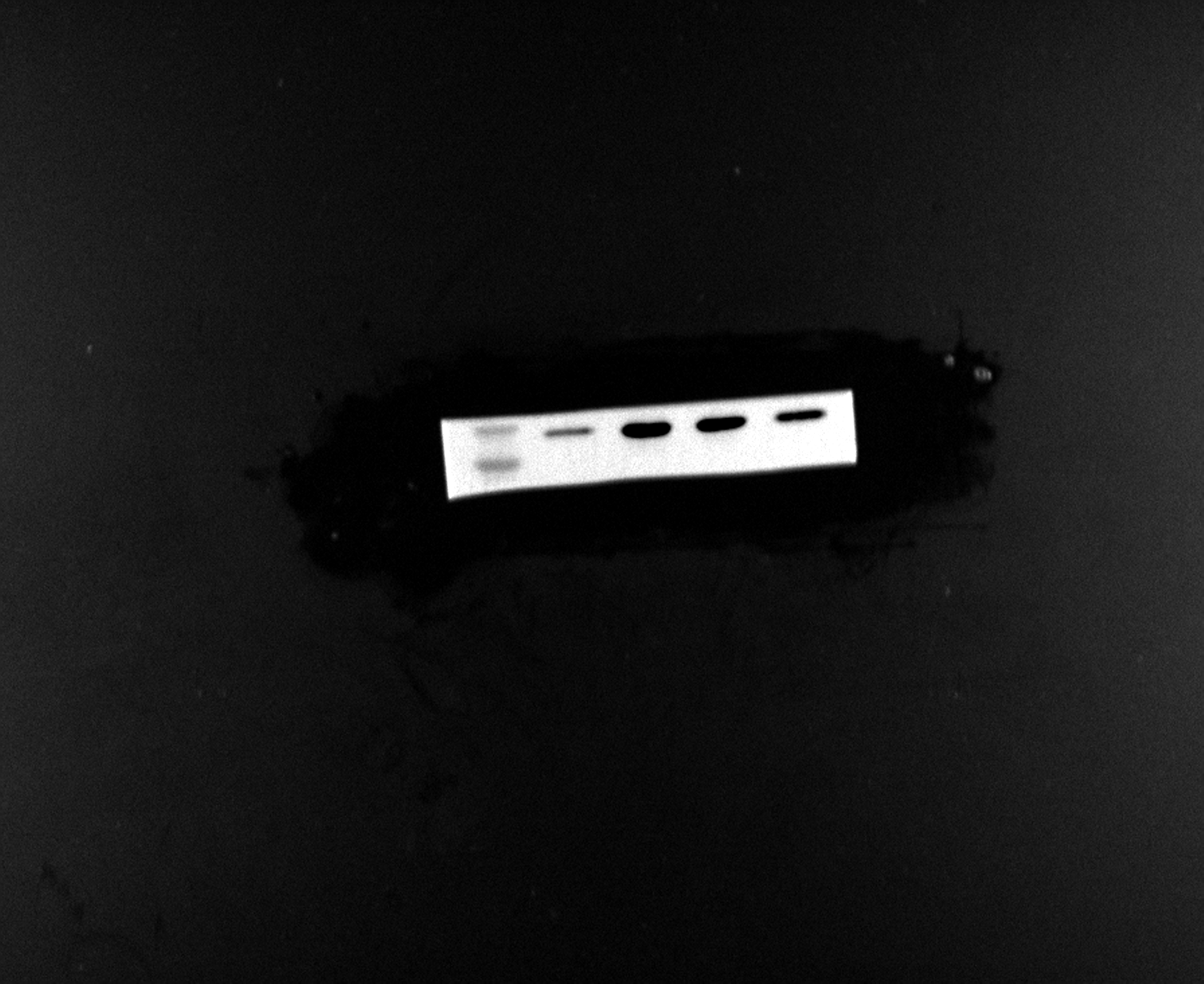

Supplement: Supplemental Information 1 [file peerj-09-11482-s001.zip › Western blot Figure/Fig2c-cleaved caspase-3.Tif]

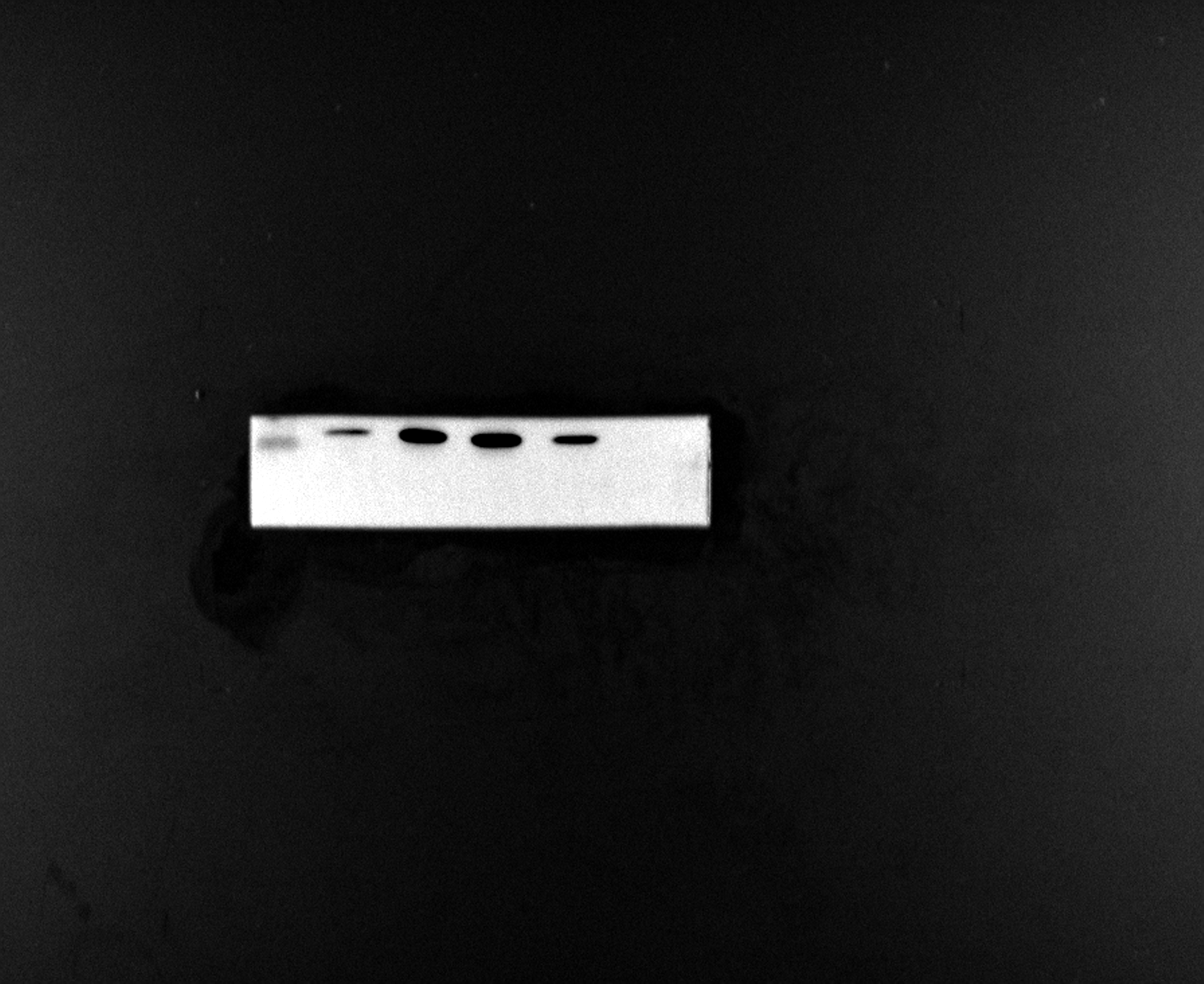

Supplement: Supplemental Information 1 [file peerj-09-11482-s001.zip › Western blot Figure/Fig2c-cleaved caspase-9.Tif]

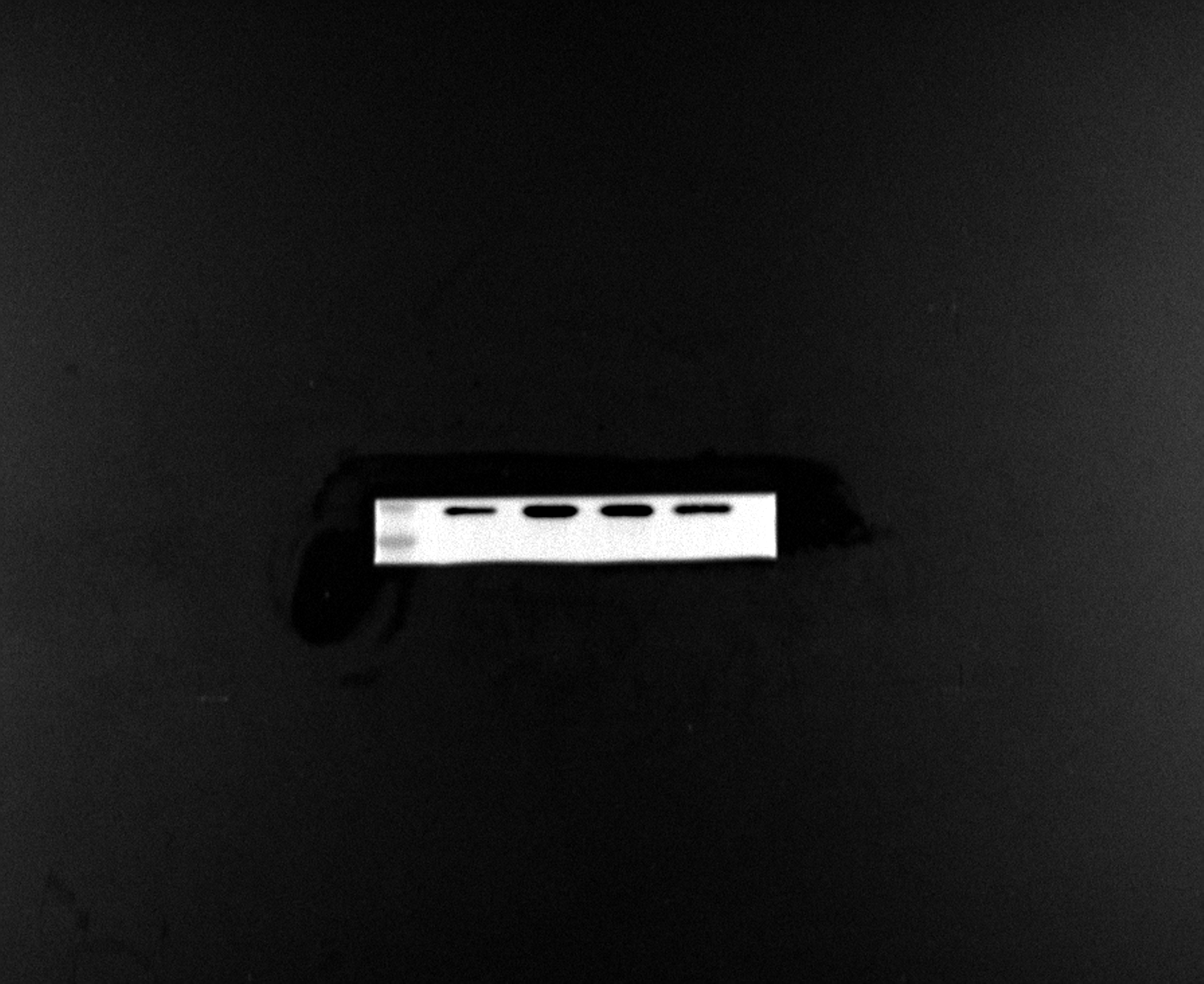

Supplement: Supplemental Information 1 [file peerj-09-11482-s001.zip › Western blot Figure/Fig2c-cytochrome c.Tif]

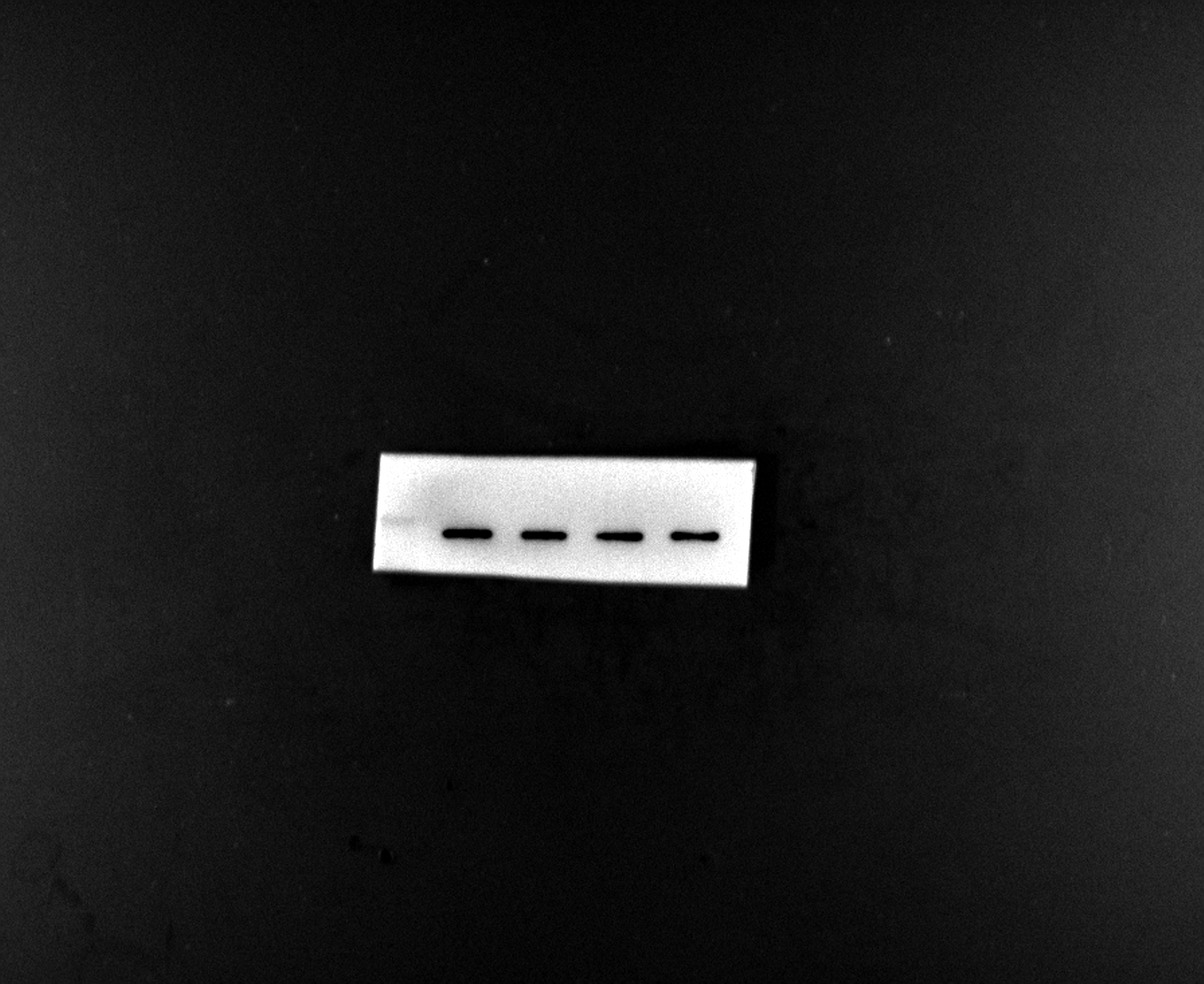

Supplement: Supplemental Information 1 [file peerj-09-11482-s001.zip › Western blot Figure/Fig2c-a┬-actin.Tif]

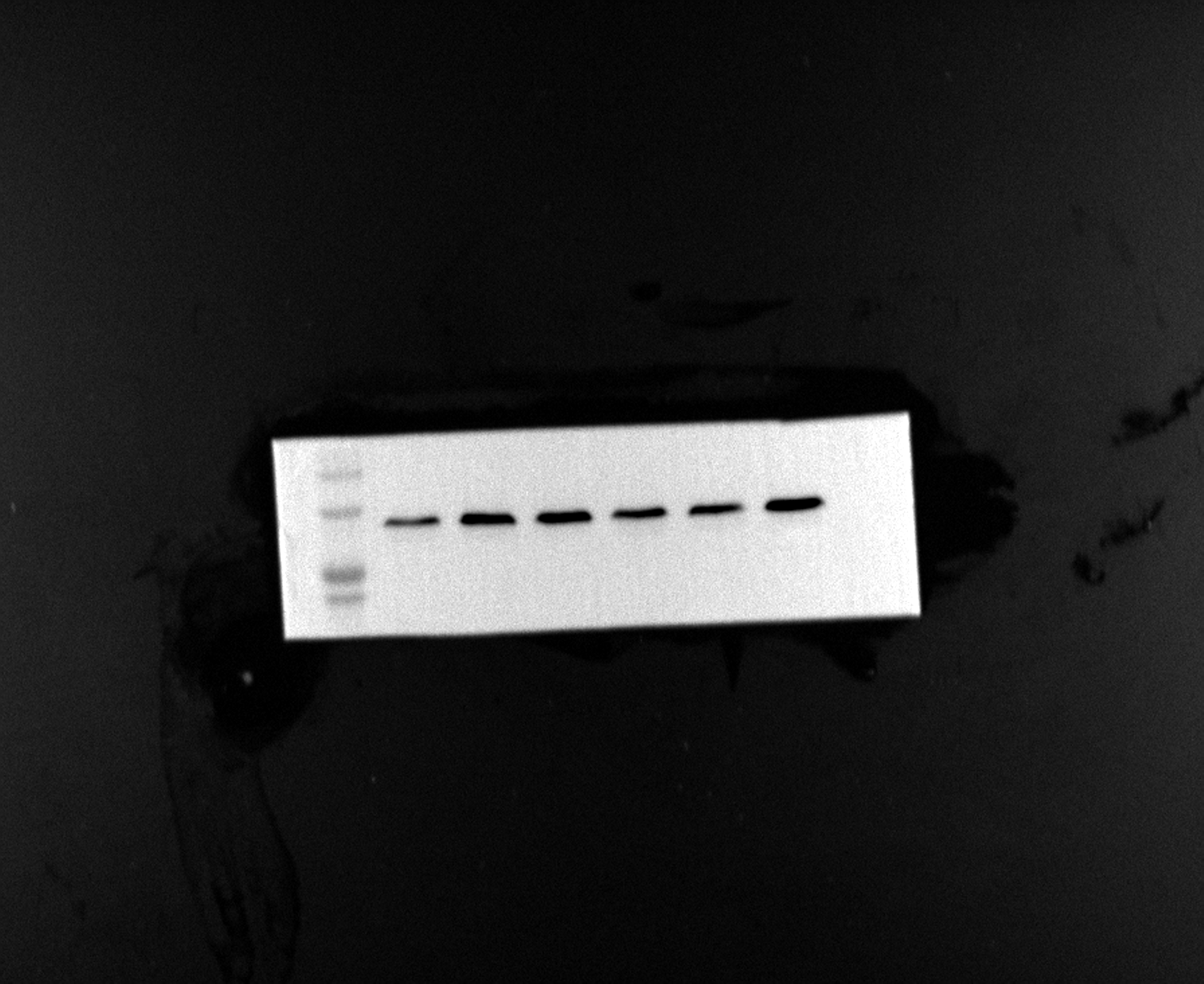

Supplement: Supplemental Information 1 [file peerj-09-11482-s001.zip › Western blot Figure/Fig5c-Bax.Tif]

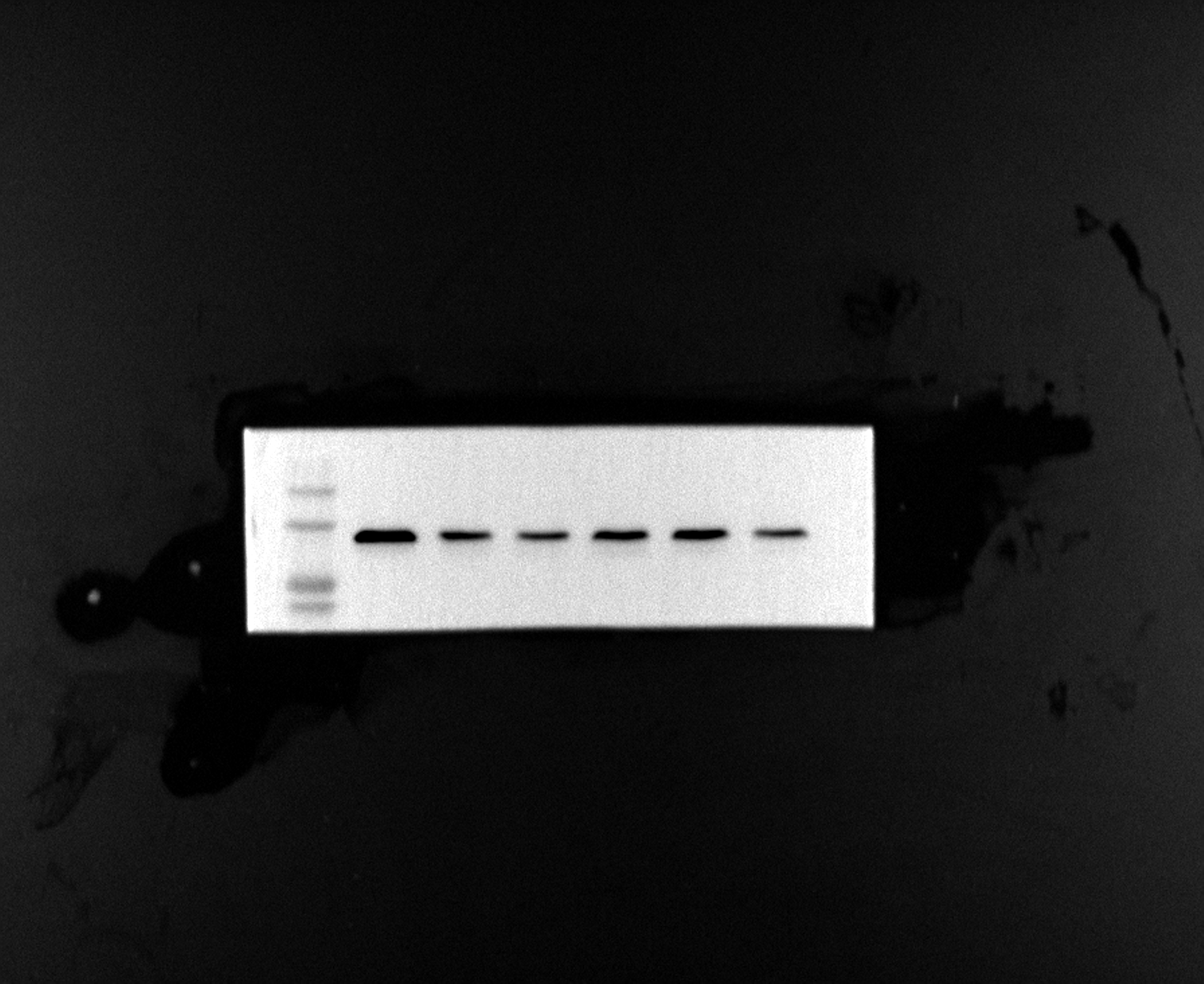

Supplement: Supplemental Information 1 [file peerj-09-11482-s001.zip › Western blot Figure/Fig5c-Bcl-2.Tif]

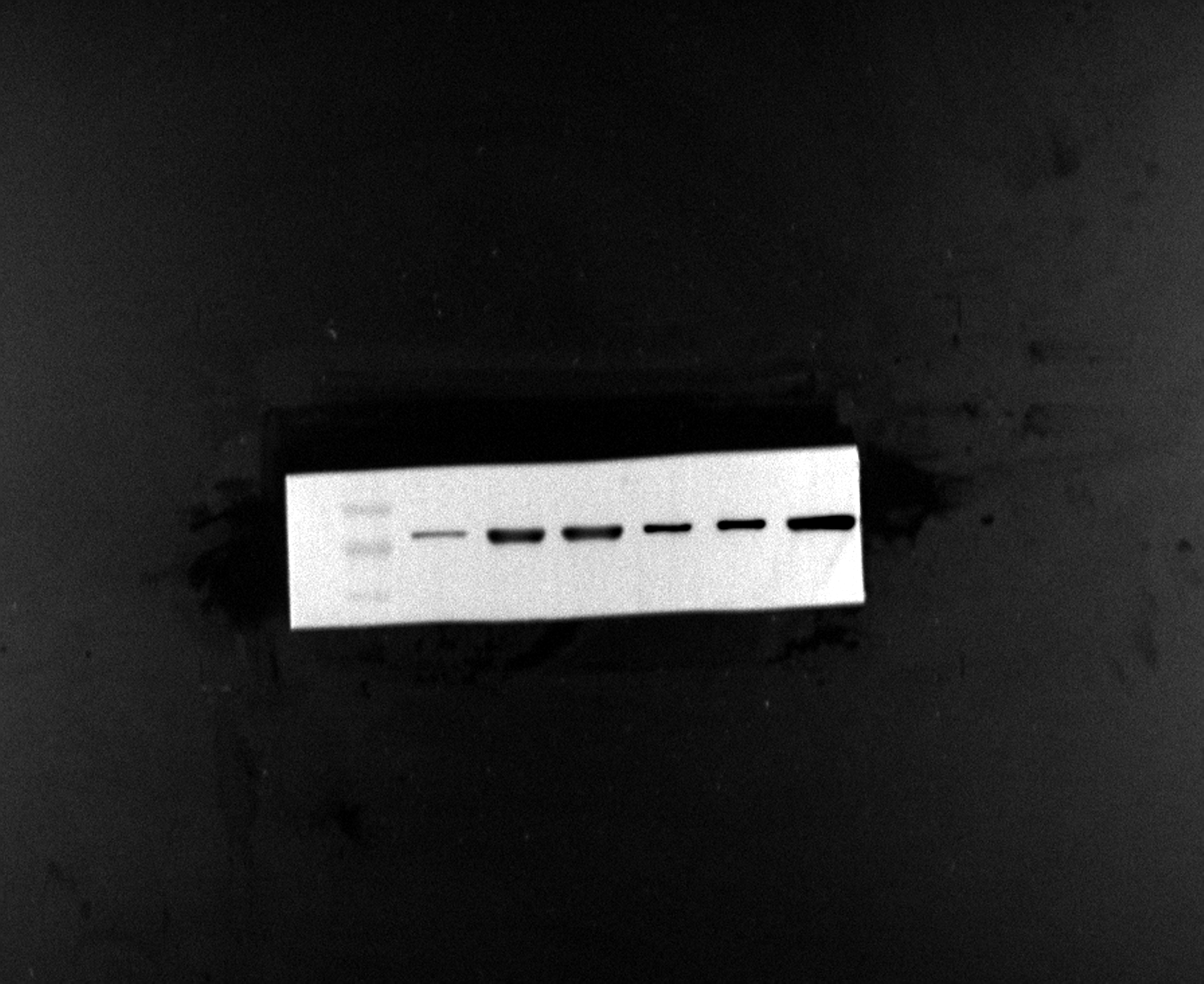

Supplement: Supplemental Information 1 [file peerj-09-11482-s001.zip › Western blot Figure/Fig5c-cleaved caspase-3.Tif]

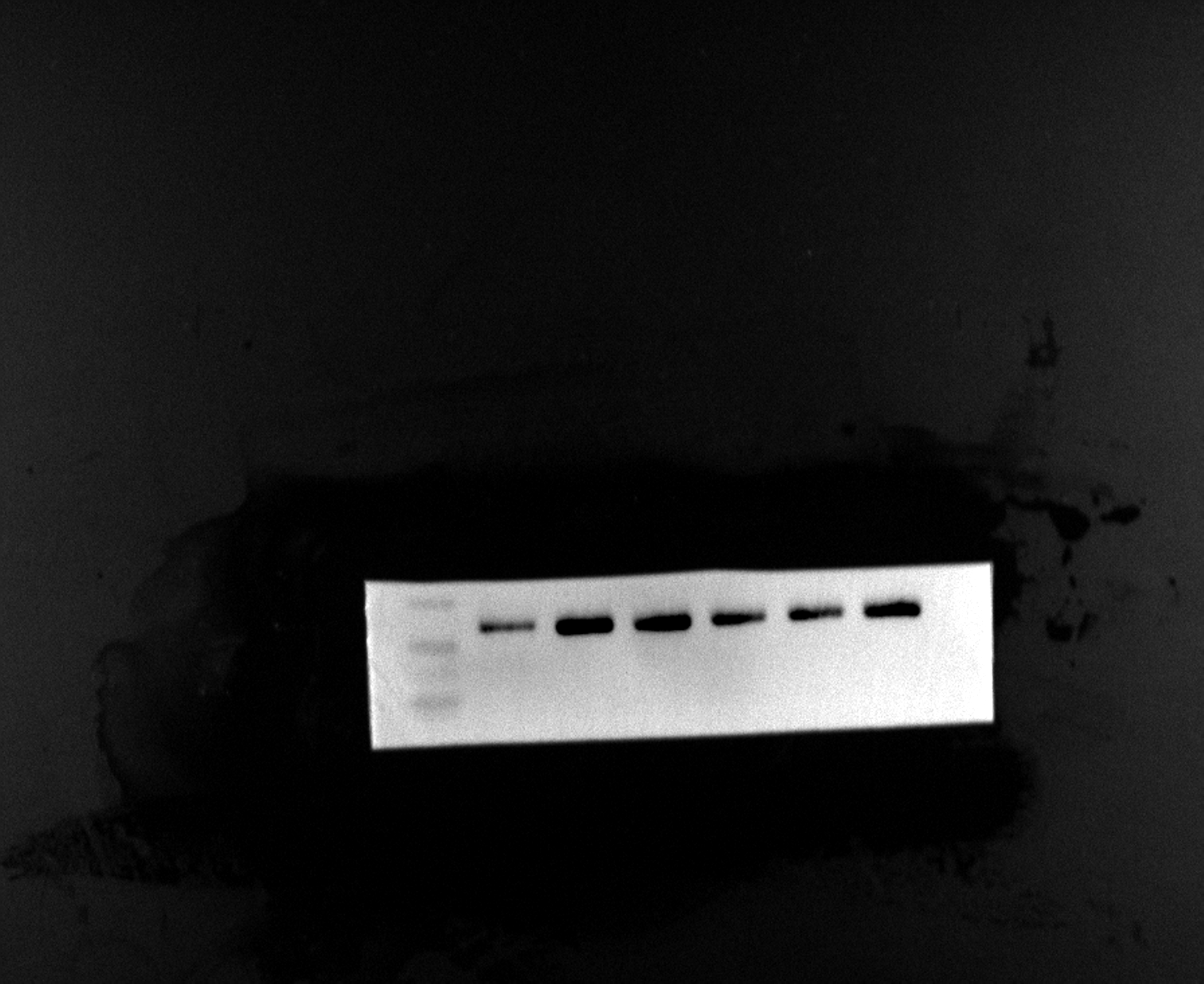

Supplement: Supplemental Information 1 [file peerj-09-11482-s001.zip › Western blot Figure/Fig5c-cleaved caspase-9.Tif]

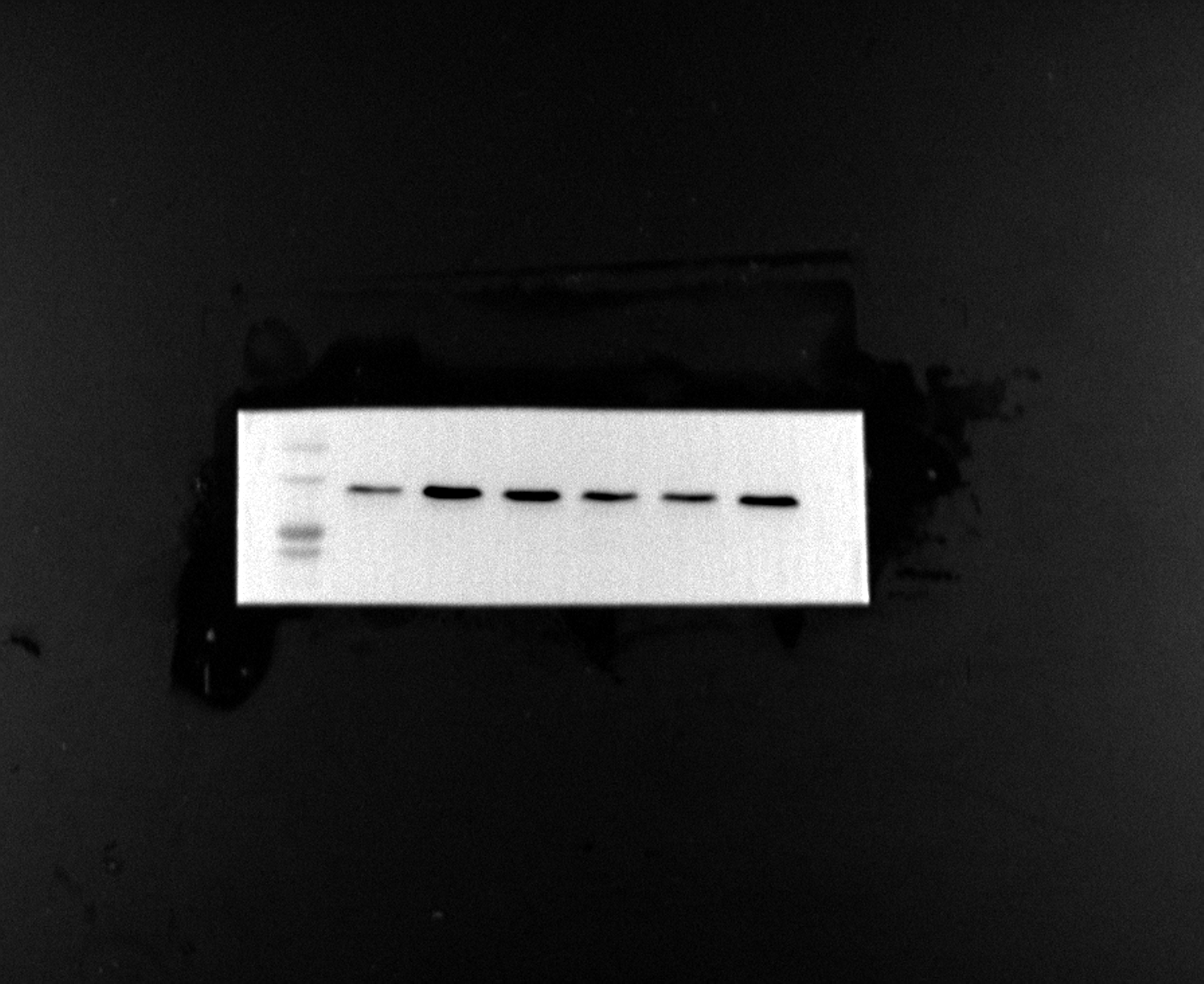

Supplement: Supplemental Information 1 [file peerj-09-11482-s001.zip › Western blot Figure/Fig5c-cytochrome c.Tif]

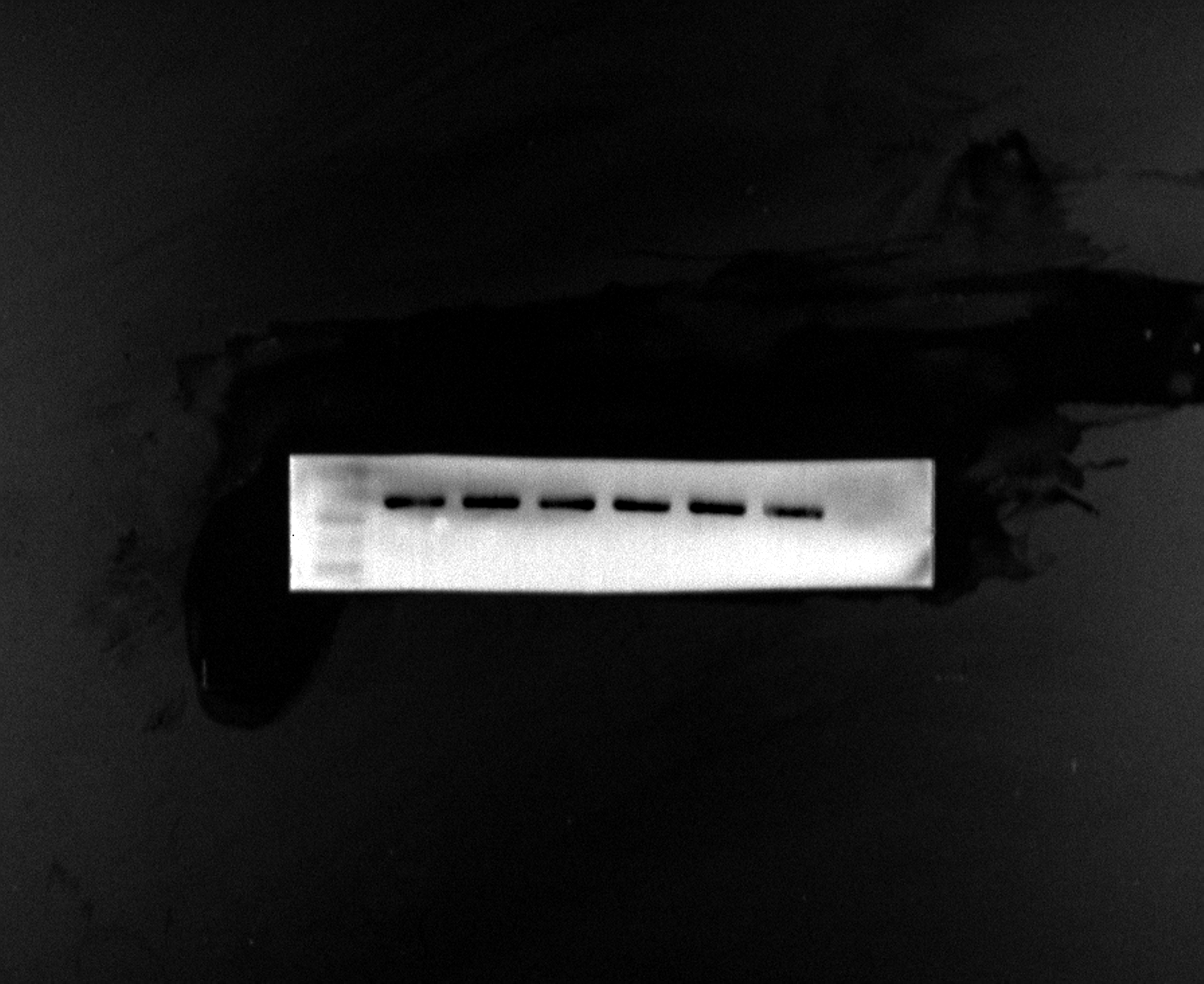

Supplement: Supplemental Information 1 [file peerj-09-11482-s001.zip › Western blot Figure/Fig5c-a┬-actin.Tif]

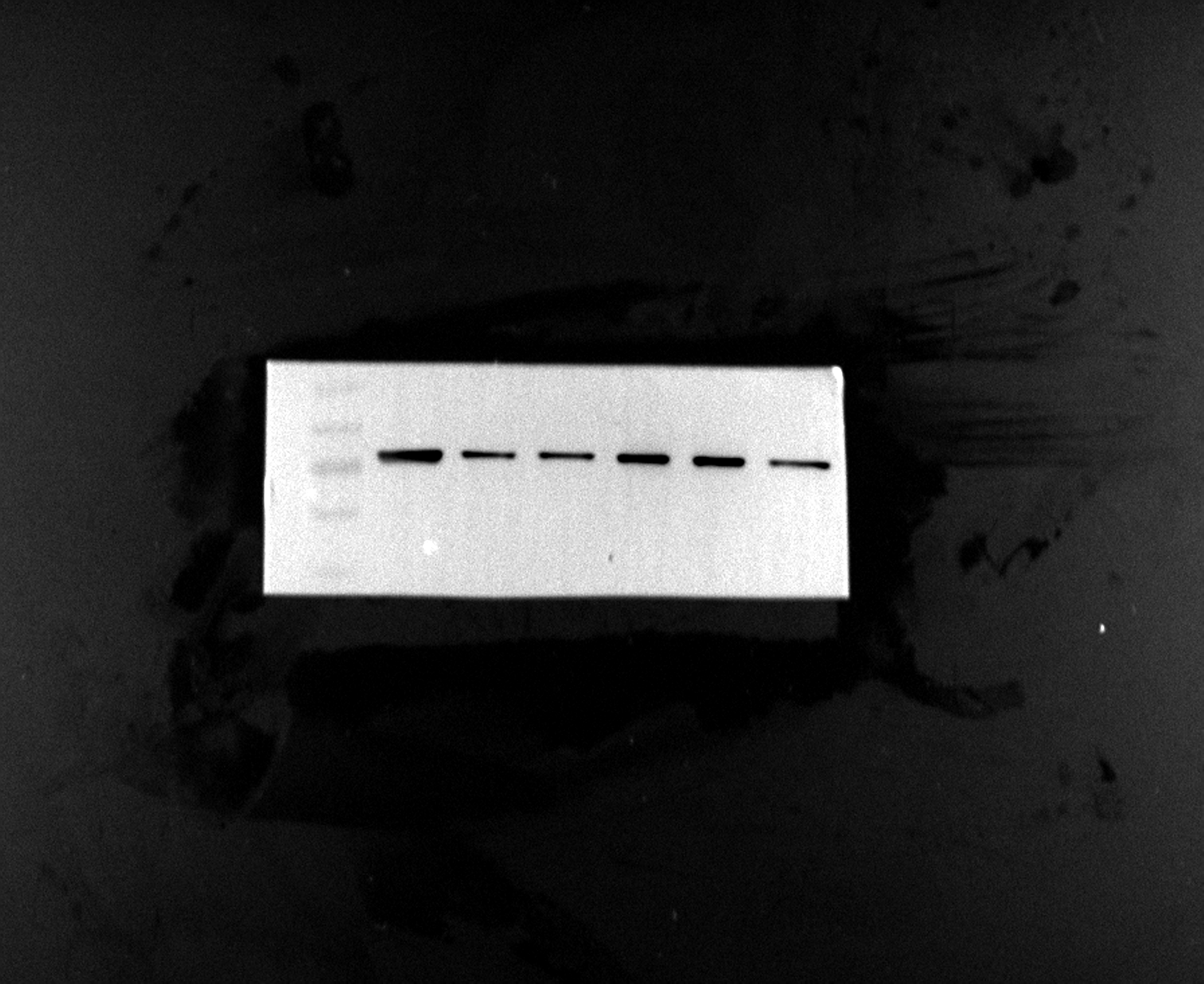

Supplement: Supplemental Information 1 [file peerj-09-11482-s001.zip › Western blot Figure/Fig7a-p-AKT.Tif]

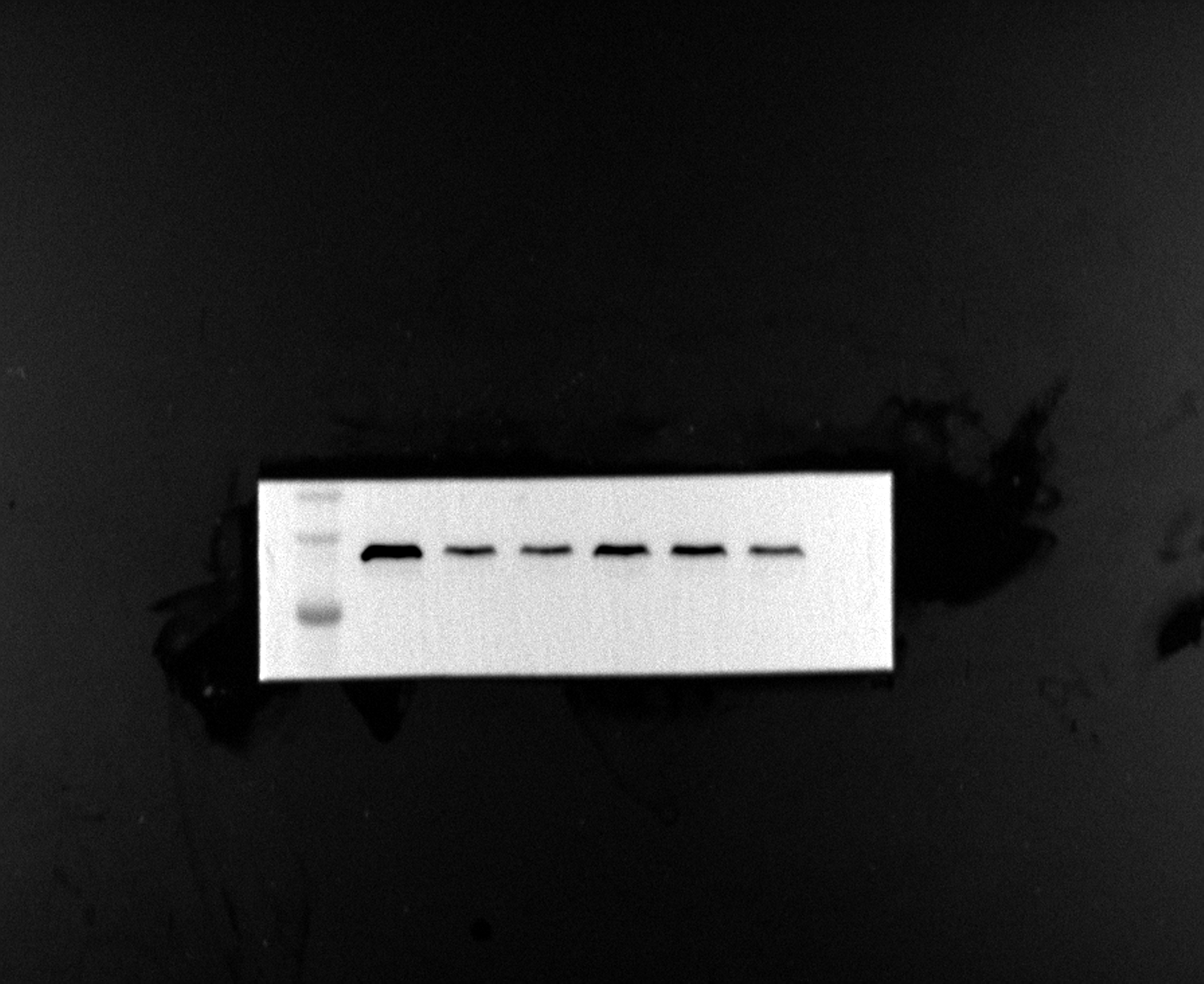

Supplement: Supplemental Information 1 [file peerj-09-11482-s001.zip › Western blot Figure/Fig7a-p-PI3K.Tif]

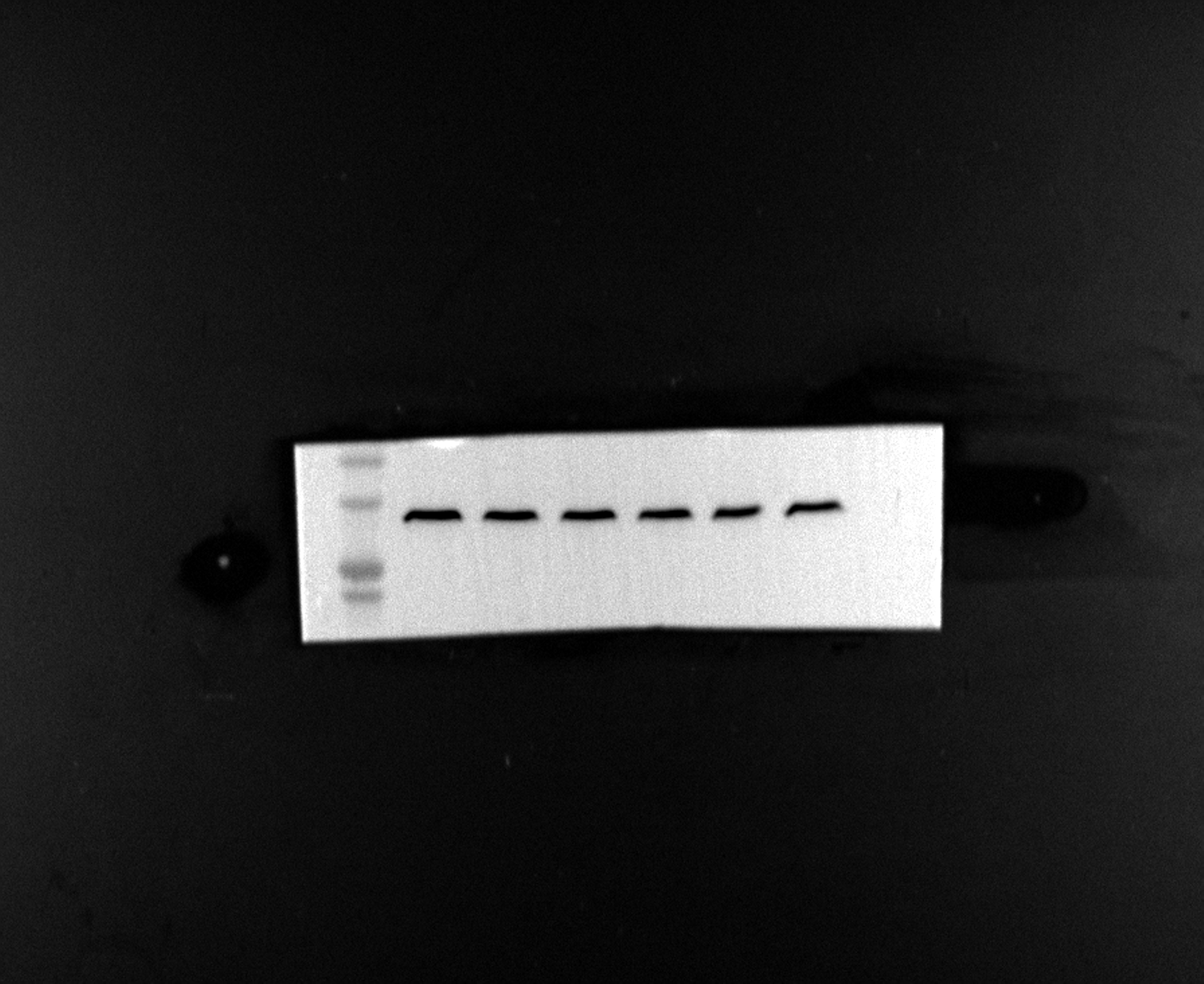

Supplement: Supplemental Information 1 [file peerj-09-11482-s001.zip › Western blot Figure/Fig7a-t-AKT.Tif]

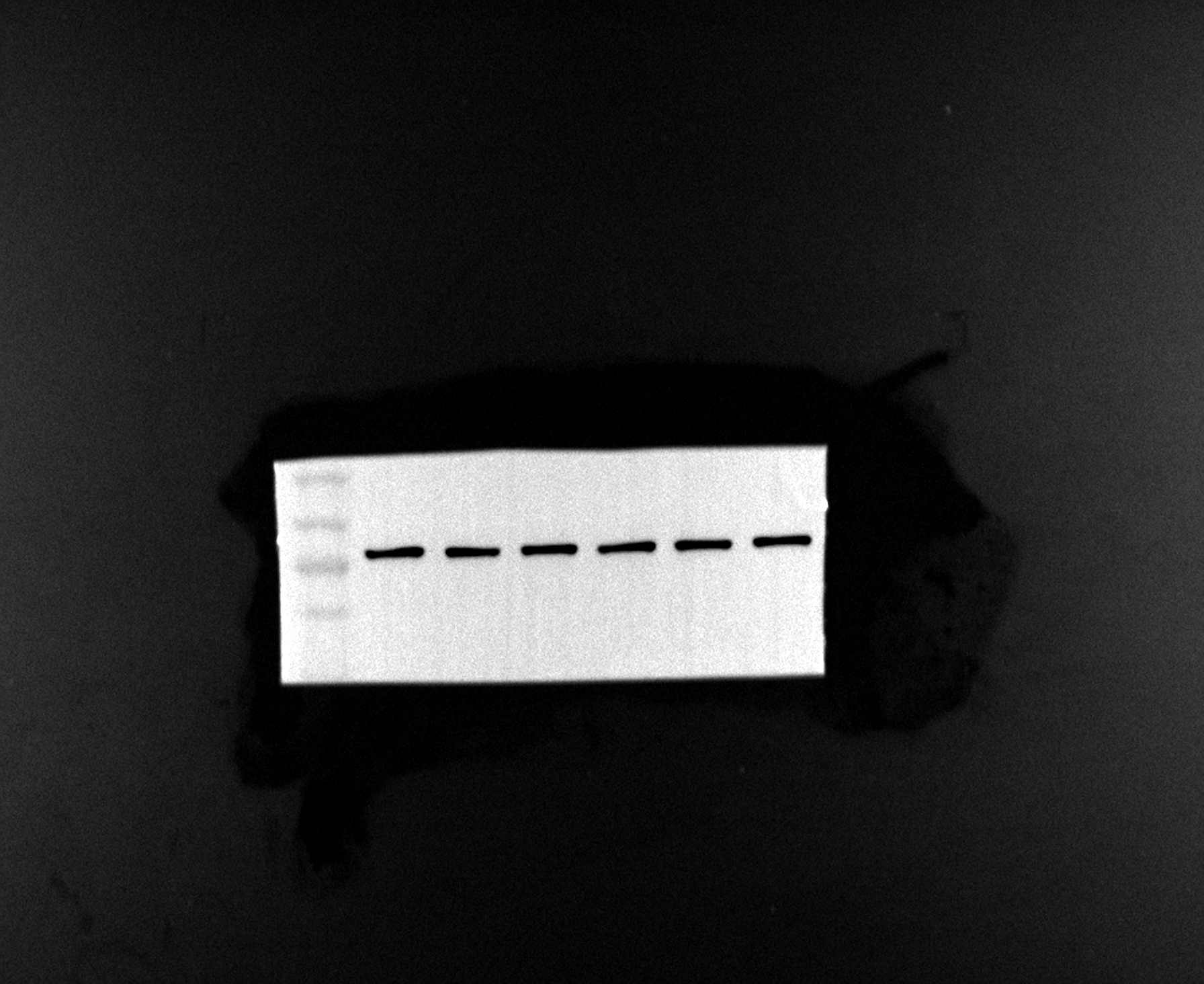

Supplement: Supplemental Information 1 [file peerj-09-11482-s001.zip › Western blot Figure/Fig7a-t-PI3K.Tif]

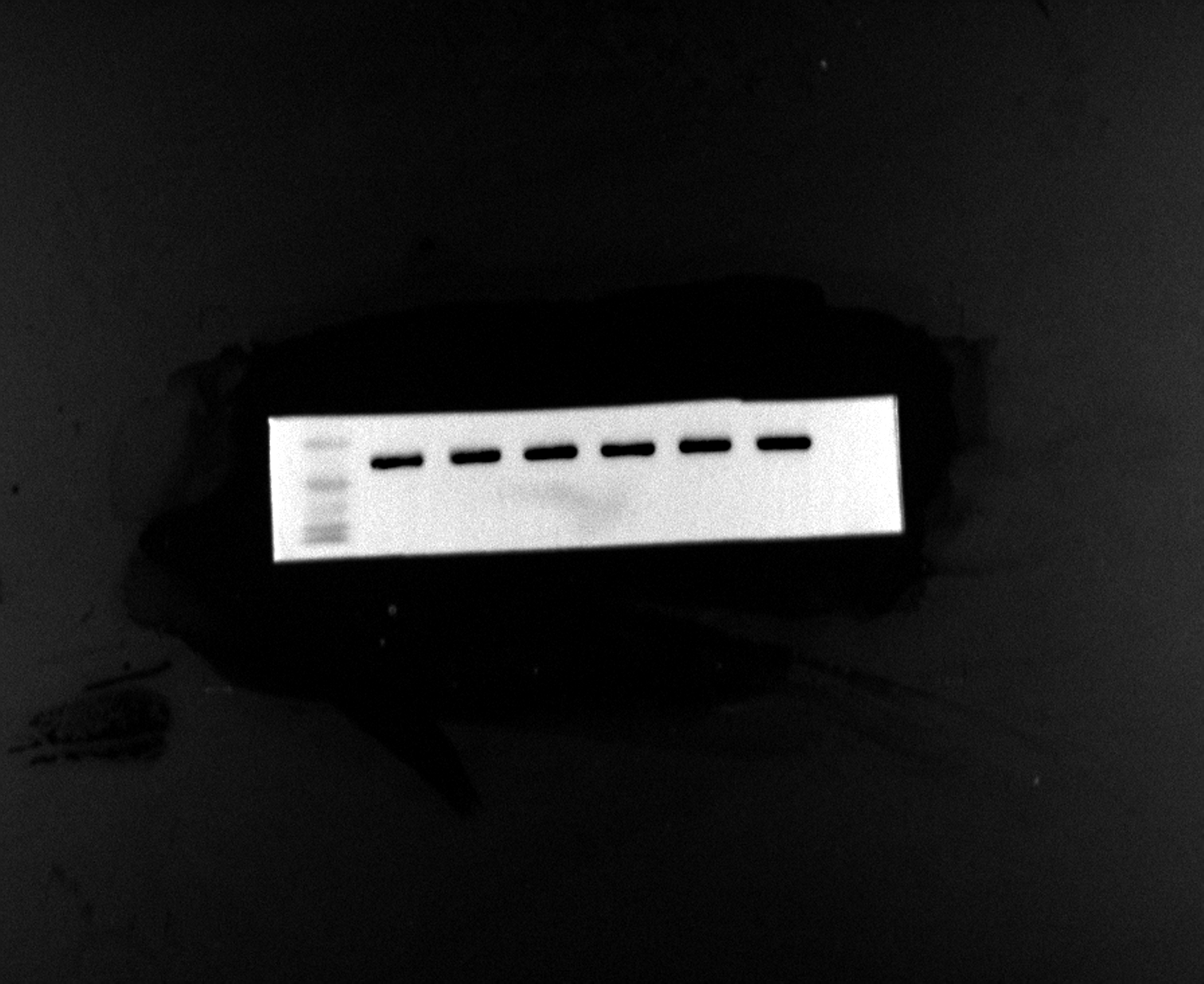

Supplement: Supplemental Information 1 [file peerj-09-11482-s001.zip › Western blot Figure/Fig7a-a┬-actin.Tif]

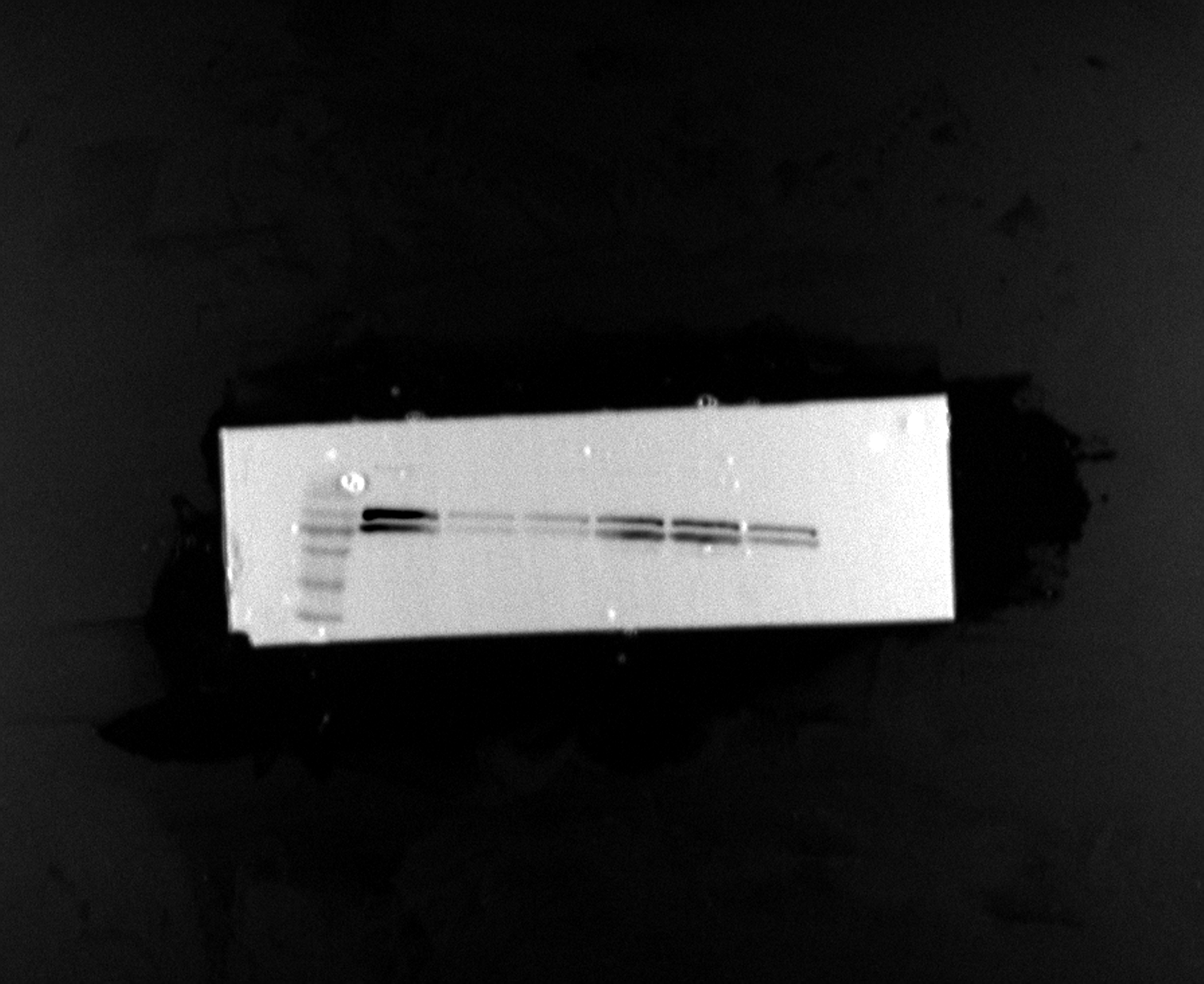

Supplement: Supplemental Information 1 [file peerj-09-11482-s001.zip › Western blot Figure/Fig7c-p-ERK1 2.Tif]

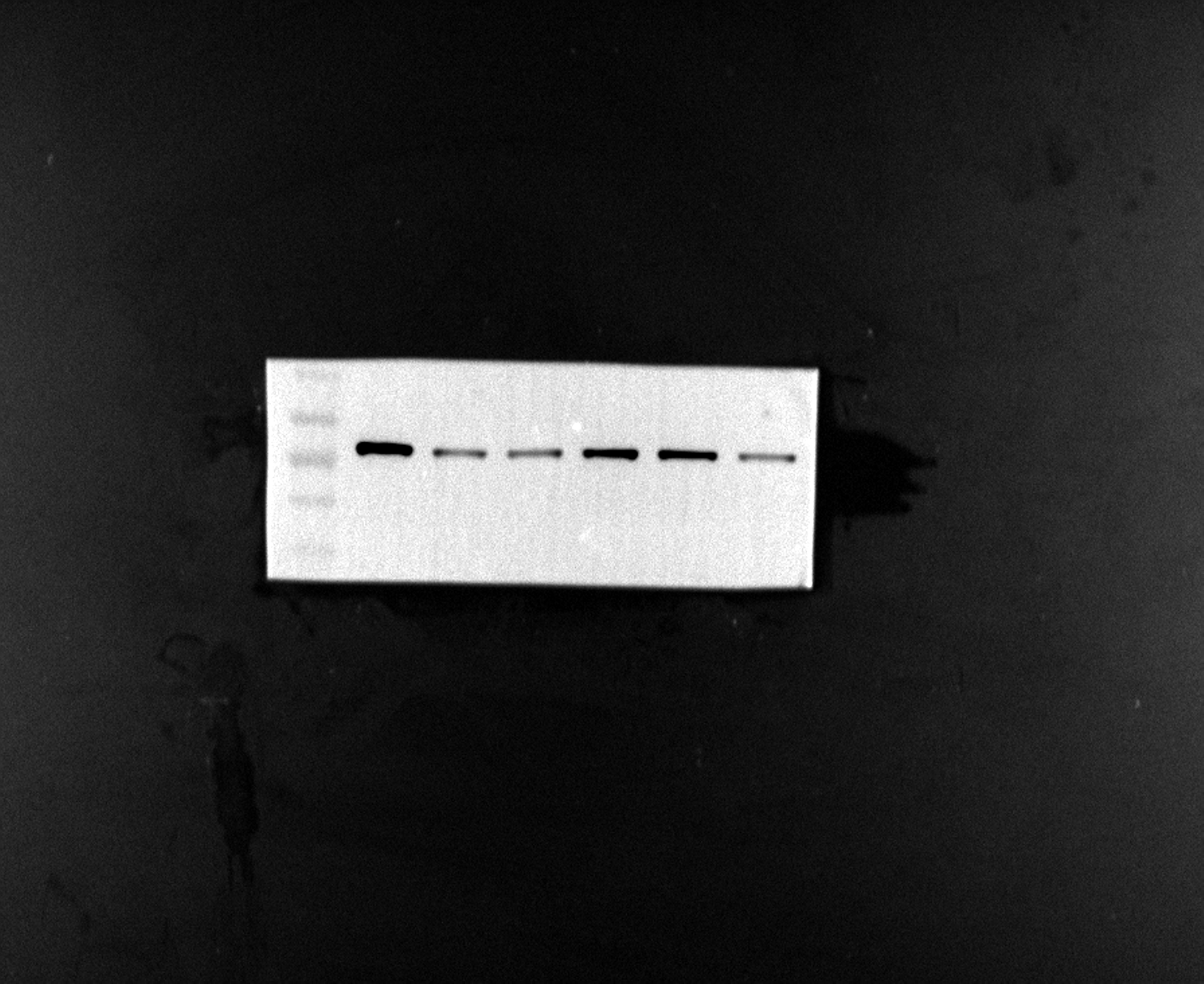

Supplement: Supplemental Information 1 [file peerj-09-11482-s001.zip › Western blot Figure/Fig7c-p-MEK1.Tif]

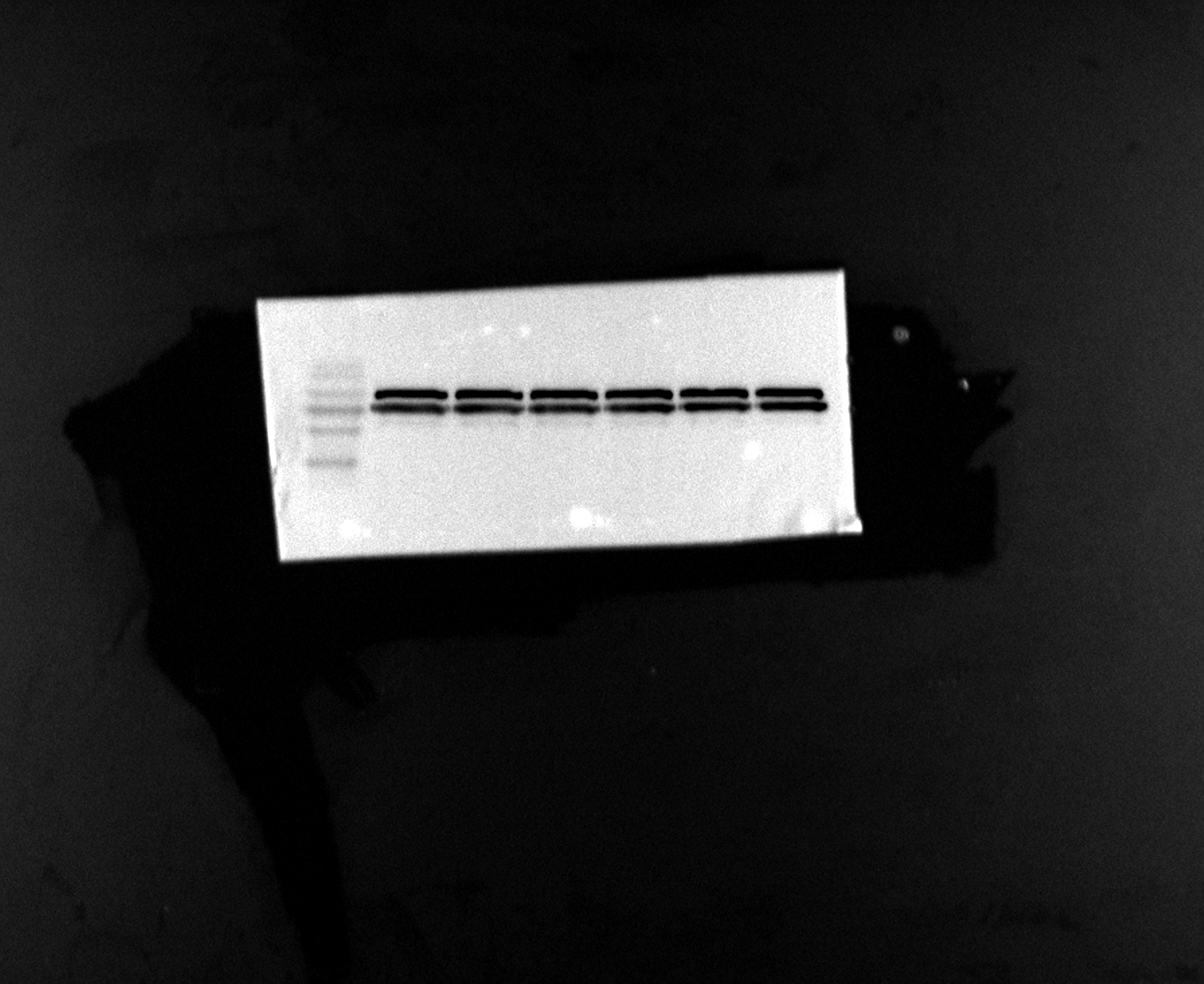

Supplement: Supplemental Information 1 [file peerj-09-11482-s001.zip › Western blot Figure/Fig7c-t-ERK 1 2.Tif]

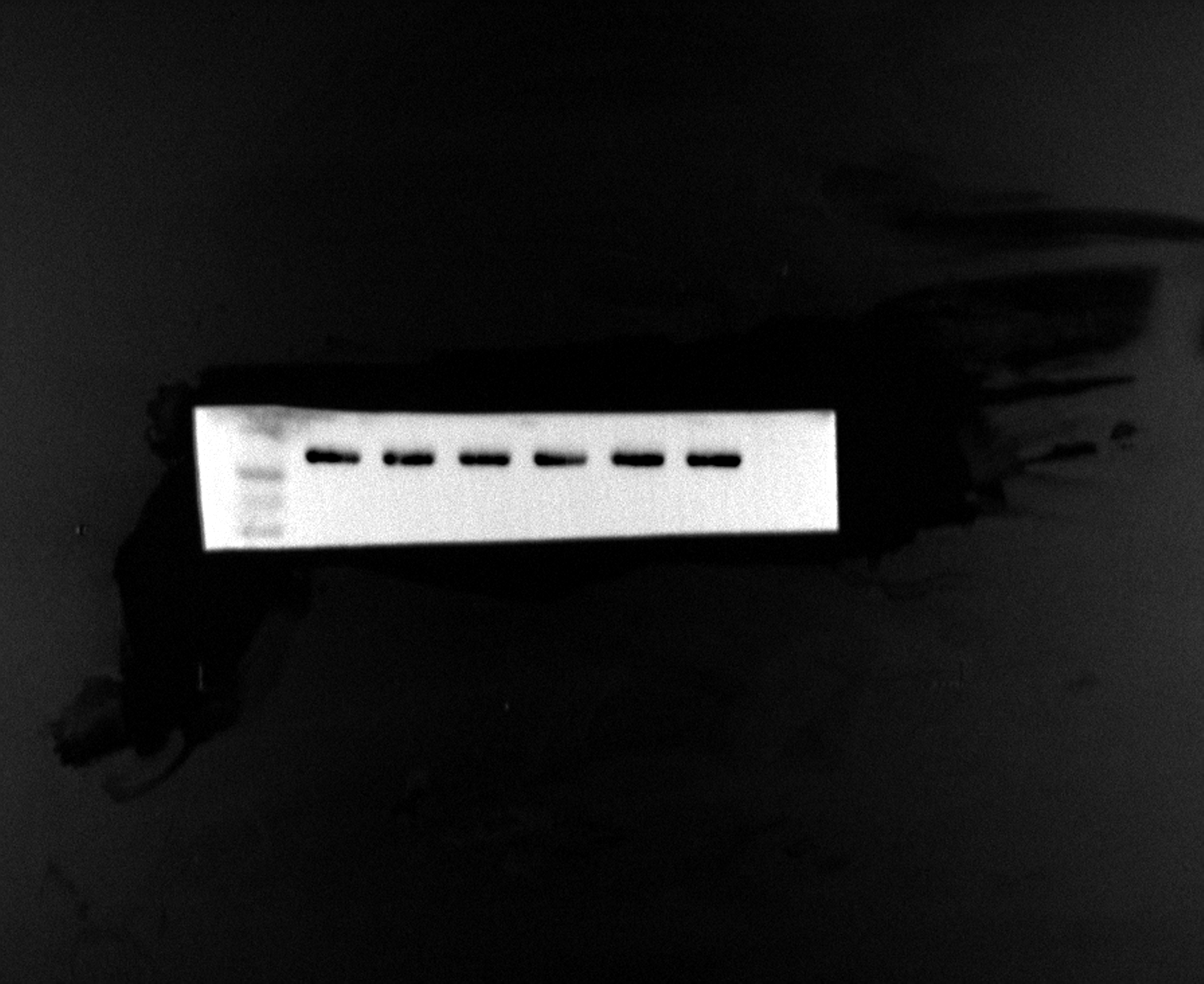

Supplement: Supplemental Information 1 [file peerj-09-11482-s001.zip › Western blot Figure/Fig7c-t-MEK1.Tif]

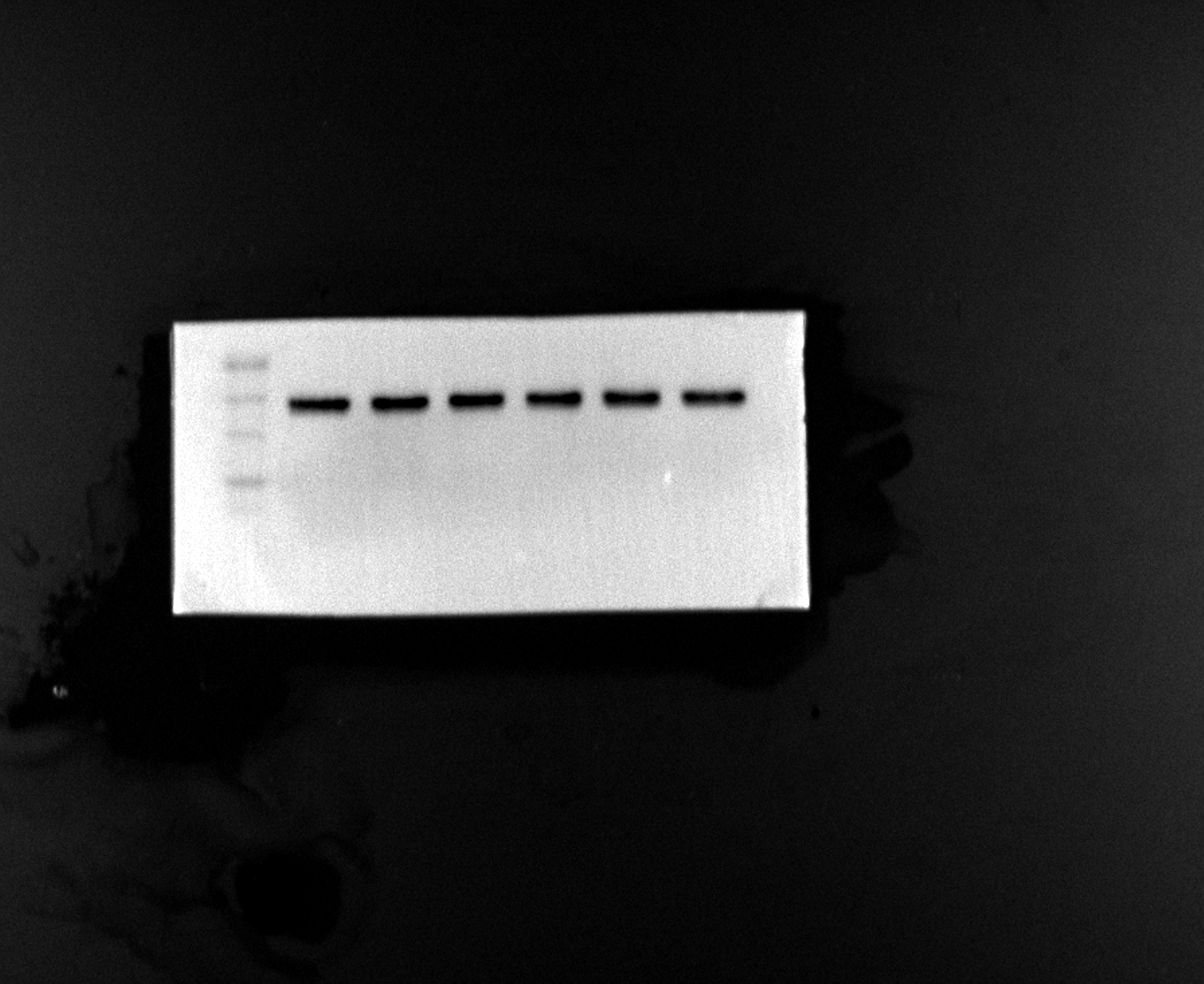

Supplement: Supplemental Information 1 [file peerj-09-11482-s001.zip › Western blot Figure/Fig7c-a┬-actin.Tif]
